# Identification of antigenic domains and peptides from VP15 of white spot syndrome virus and their antiviral effects in *Marsupenaeus japonicus*

**DOI:** 10.1038/s41598-021-92002-8

**Published:** 2021-06-17

**Authors:** Jirayu Boonyakida, Jian Xu, Jun Satoh, Takafumi Nakanishi, Tohru Mekata, Tatsuya Kato, Enoch Y. Park

**Affiliations:** 1 Department of Bioscience, Graduate School of Science and Technology, Shizuoka University, 836 Ohya, Suruga-ku, Shizuoka 422-8529 Japan; 2 Institute of Biology and Information Science, Biomedical Synthetic Biology Research Center, School of Life Sciences, East China Normal University, Shanghai, 200062 People’s Republic of China; 3 Fisheries Technology Institute of National Research and Development Agency, Japan Fisheries Research and Education Agency, Tamaki Field Station, Mie 519-0423 Japan; 4 Department of Applied Biological Chemistry, Graduate School of Integrated Science and Technology, Shizuoka University, 836 Ohya, Suruga-ku, Shizuoka 422-8529 Japan; 5 Fisheries Technology Institute of National Research and Development Agency, Japan Fisheries Research and Education Agency, Namsei Field Station, Mie 516-0193 Japan; 6 Research Institute of Green Science and Technology, Shizuoka University, 836 Ohya, Suruga-ku, Shizuoka 422-8529 Japan

**Keywords:** Biotechnology, Animal biotechnology, Expression systems

## Abstract

White spot syndrome virus (WSSV) is one of the most devastating pathogens in penaeid shrimp and can cause massive damage in shrimp aquaculture industries. Previously, the WSSV structural protein VP15 was identified as an antigenic reagent against WSSV infections. In this study, we truncated this protein into VP15_(1–25)_, VP15_(26–57)_, VP15_(58–80)_, and VP15_(1–25,58–80)_. The purified proteins from the *E. coli* expression system were assayed as potential protective agents in Kuruma shrimp (*Marsupenaeus japonicus*) using the prime-and-boost strategy. Among the four truncated constructs, VP15_(26–57)_ provided a significant improvement in the shrimp survival rate after 20 days of viral infection. Subsequently, four peptides (KR11, SR11, SK10, and KK13) from VP15_(26–57)_ were synthesized and applied in an in vivo assay. Our results showed that SR11 could significantly enhance the shrimp survival rate, as determined from the accumulated survival rate. Moreover, a multiligand binding protein with a role in the host immune response and a possible VP15-binding partner, MjgC1qR, from the host *M. japonicus* were employed to test its binding with the VP15 protein. GST pull-down assays revealed that MjgC1qR binds with VP15, VP15_(26–57)_, and SR11. Taken together, we conclude that SR11 is a determinant antigenic peptide of VP15 conferring antiviral activity against WSSV.

## Introduction

White spot disease (WSD), caused by white spot syndrome virus (WSSV), has been known as one of the most devastating diseases of farmed shrimp worldwide since its first occurrence in Taiwan in 1992^1–3^. Since then, substantial global losses due to WSD alone have been approximately US$15 billion annually, with an increasing rate of US$1 billion/year^4,5^. WSSV infection in a shrimp pond could result in a high mortality rate within a single week, especially in penaeid shrimp (*e.g., Marsupenaeus japonicus*, *Litopenaeus vannamei*, *Penaeus monodon*, and *Fenneropenaeus indicus*)^6,7^. Clinical signs in WSD-suffering shrimp include lethargy, anorexia, reduction in food uptake, reduced preening activities, loose cuticle, reddish discoloration, and white calcified spots of 0.5–3 mm in diameter on the exoskeleton^8,9^.

WSSV is the sole member of the genus *Whispovirus* and the only genus in the *Nimaviridae* family^10–12^. The virus is a large, enveloped virus containing supercoiled circular double-stranded DNA (dsDNA) with an unknown functional tail-like appendage^12,13^. The virion has a size of approximately 80–100 × 250–350 nm with a rod-shaped nucleocapsid covered by a trilaminar membrane^14^. The genomic DNA is 285–305 kbp long with nine tandem repeat conserved regions and 180 putative open reading frames (ORFs). However, most of the ORF-encoded proteins have no homology to the known proteins in databases^15–18^. Proteomic analysis of WSSV revealed that the virus contains at least six major virion proteins (VPs). VP19, VP24, VP26, and VP28 were identified as envelope proteins, while VP15 and VP664 were identified as nucleocapsid-associated proteins^19,20^. There is evidence that envelope proteins can form a protein complex that plays a crucial role in host-virus interactions^21–23^. Although, multicellular organisms possess an ability to recognize and protect their selves against pathogenic bacteria and viruses called “immune system”, invertebrates such as insects and crustaceans do not own a long-term immunity of adaptive immune system. Therefore, their defensive mechanisms totally depend on the innate immune system^24,25^. The innate immune system is initiated when pathogen-associated molecular patterns (PAMPs) from pathogens are being recognized by host pattern recognition receptors (PRRs)^26,27^. Then a series of signaling pathways of humoral and cellular responses is stimulated, thus molecules such as antimicrobial peptides (AMPs), phenoloxidase (PO), and lysozymes are produced consequently or enhance cellular responses such as phagocytosis, clotting, or apoptosis^26–30^.

However, there is no practical method to prevent WSSV infection in shrimp and to manage the spread of this disease. Hence, it is necessary to develop a treatment against the virus. To date, following the concept of “trained immunity”, several kinds of WSSV immunizing agents have been tested, including viral protein subunits, attenuated WSSV, and DNA-/RNA-based agents^31,32^. Most recombinant subunit vaccines investigated have been based on VP19, VP24, VP26, VP28, VP292, and VP466, and among them, VP28 has been widely studied^33–37^. These viral subunits were confirmed to improve the survival rate after challenging shrimp with WSSV. Recently, we reported that VP15 can also provide a protective effect in Kuruma shrimp (*M. japonicus*) against WSSV after immune prime-and-boost via intramuscular injection^38^.

WSSV-VP15 is an 80 amino acid protein with an extremely high basic pI value of 12.49, only showing homology with the DNA-binding proteins of eukaryotic origin and a baculovirus p6.9 protein^39^. Some studies have suggested that VP15 may be involved in viral genome packaging into the capsid and a major nucleocapsid protein with the ability to self-interact, forming homomultimers^40,41^. Although VP15 is a major nucleocapsid protein, its properties and functions have not been defined. No protein crystal structure has been reported yet, and only one trial used VP15-based material (DNA vaccine encoding VP15^42^) for immunizing shrimp against WSSV. We have previously demonstrated that VP15 can enhance shrimp survivability after challenge with WSSV^38^. In this study, we attempted to determine the antigenic domain of the VP15 protein by in vivo animal experiments with *M. japonicus* using a purified truncated VP15 series and synthetic peptides derived from VP15 at the peptide level. To further explore the mechanism of antigenicity involved, we also investigated the possible interaction between VP15 and its host protein partner, a gC1qR homolog from *M. japonicus* (MjgC1qR), via GST pull-down assay.

## Results

### Expression and purification of truncated WSSV-VP15s

**Figure 1:**
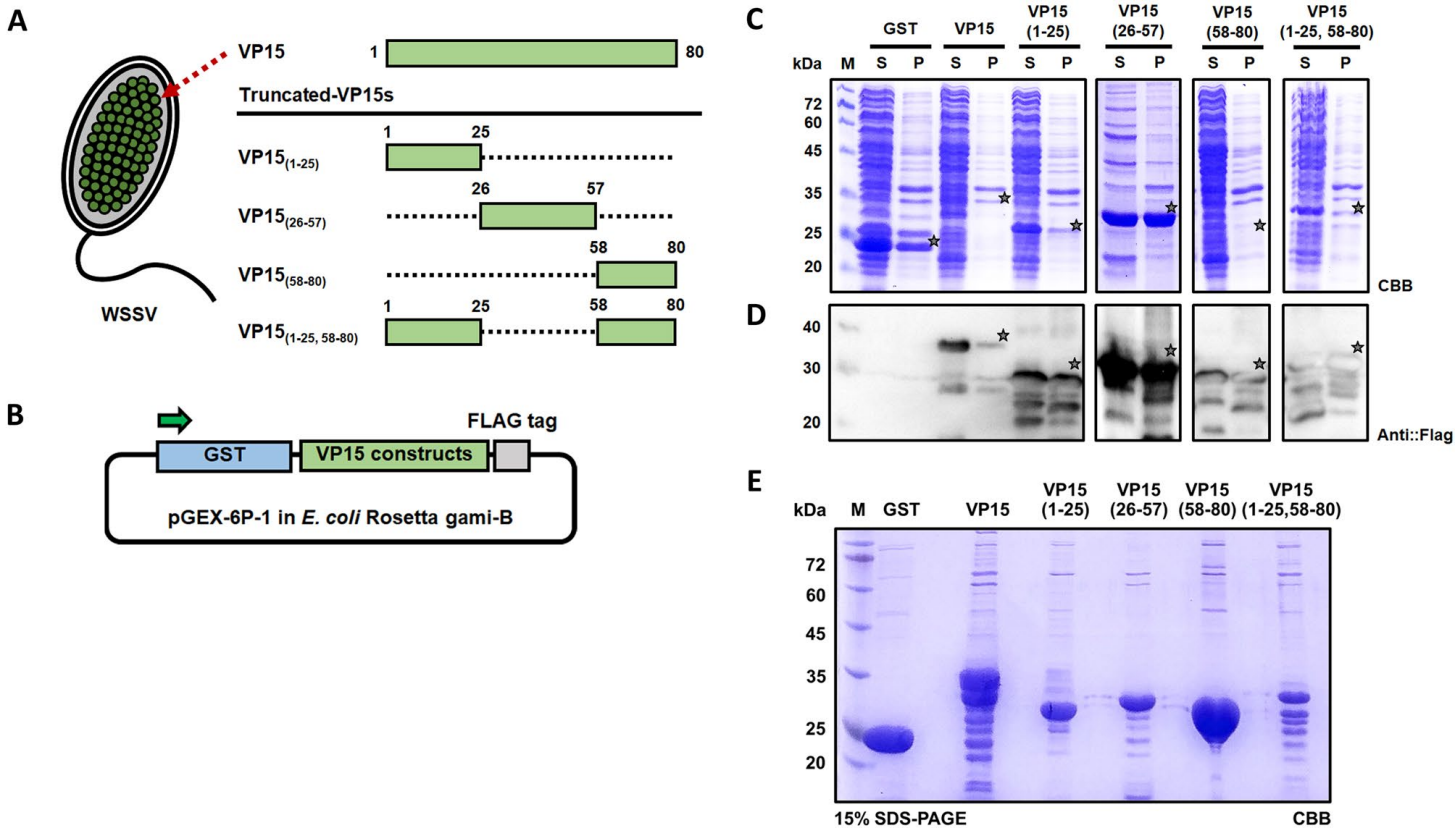
Expression of the truncated WSSV-VP15 series using the *E. coli* expression system. (**A**) Schematic diagram representing a major nucleocapsid protein of WSSV-VP15. Four truncated VP15 constructs were generated as illustrated and were named VP15_(1–25)_, VP15_(26–57)_, VP15_(58–80)_, and VP15_(1–25,58–80)_. The numbers represent the amino acid position. (**B**) Construction of plasmids harboring the VP15 truncated series for expression using the *E. coli* system and expression verification via SDS-PAGE. GST: glutathione-S-transferase as a fusion tag and for affinity purification; Flag: DYKDDDDK epitope tag for Western blot analysis. (**C**) Coomassie brilliant blue-stained SDS-PAGE gel of *E. coli* expressing VP15 truncated constructs and (**D**) Western blot analysis of the expressed recombinant proteins. Anti-Flag antibody was applied for the detection of the target proteins. M: marker; S: supernatant; and P: precipitate. (**E**) SDS-PAGE analysis of purified VP15 and truncated VP15 series. Each recombinant protein was purified via GST affinity chromatography. The theoretical sizes of VP15, VP15_(1–25)_, VP15_(26–57)_, VP15_(58–80)_, and VP15_(1–25,58–80)_ were 37.0, 30.8, 31.6, 30.5, and 33.4 kDa, respectively. A main purified product of VP15_(1–25)_, VP15_(26–57)_, and VP15_(58–80)_ can be observed corresponding to the estimated size of the protein with minor bands below the main product. However, a purified product of VP15 and VP15_(1–25,58–80)_ has several bands below the main product. GST was loaded as a control. M indicates a protein marker.

pGEX-6P-1, which has a glutathione S-transferase (GST, molecular weight; 26 kDa) fusion system, was genetically fused with the VP15 gene as the GST-VP15 fusion gene, and the constructed plasmid was used as a template to generate a truncated series of VP15 (Fig. 1A,B). The protein expression was driven by IPTG induction under the control of the *tac* promoter. The sizes of the GST-fused truncated VP15 series members were predicted to be 30.8, 31.6, 30.5, and 33.4 kDa for VP15_(1–25)_, VP15_(26–57)_, VP15_(58–80)_, and VP15_(1–25,58–80)_, respectively, while that of GST-VP15 was 37.0 kDa. The expression of recombinant proteins was confirmed by both SDS-PAGE (Figs. 1C, S1, S2) and Western blotting with an anti-Flag antibody (Figs. 1D, S1, S2 of Supplementary information). The proteins were then purified from *E. coli* soluble fractions using GST affinity chromatography (Fig. 1E; SDS-PAGE in Fig. S3 and Western blot in Fig. S4 of Supplementary information) The yield of purified proteins obtained from 1 L culture was 3.3 mg for GST, 5.3 mg for VP15, 5.7 mg for VP15_(1–25)_, 6.3 mg for VP15_(26–57)_, 8.4 mg for VP15_(58–80)_, and 4.0 mg for VP15_(1–25,58–80)_.

### Vaccination and challenge experiment using the truncated VP15s

The partially purified GST-fused full-length VP15 and truncated VP15 series were subsequently intramuscularly (IM)-injected into Kuruma shrimp to assess their anti-WSSV activity. Five groups were immunized twice at days 0 and 20 using purified protein at a dose of 10 µg/g shrimp and then challenged at day 10 after the second injection (Fig. 3A). Two groups (PBS and GST) were mock-treated as two independent negative controls. All groups were challenged with WSSV at a dose of 2.69 × 10^4^ DNA copies/shrimp.

**Figure 2:**
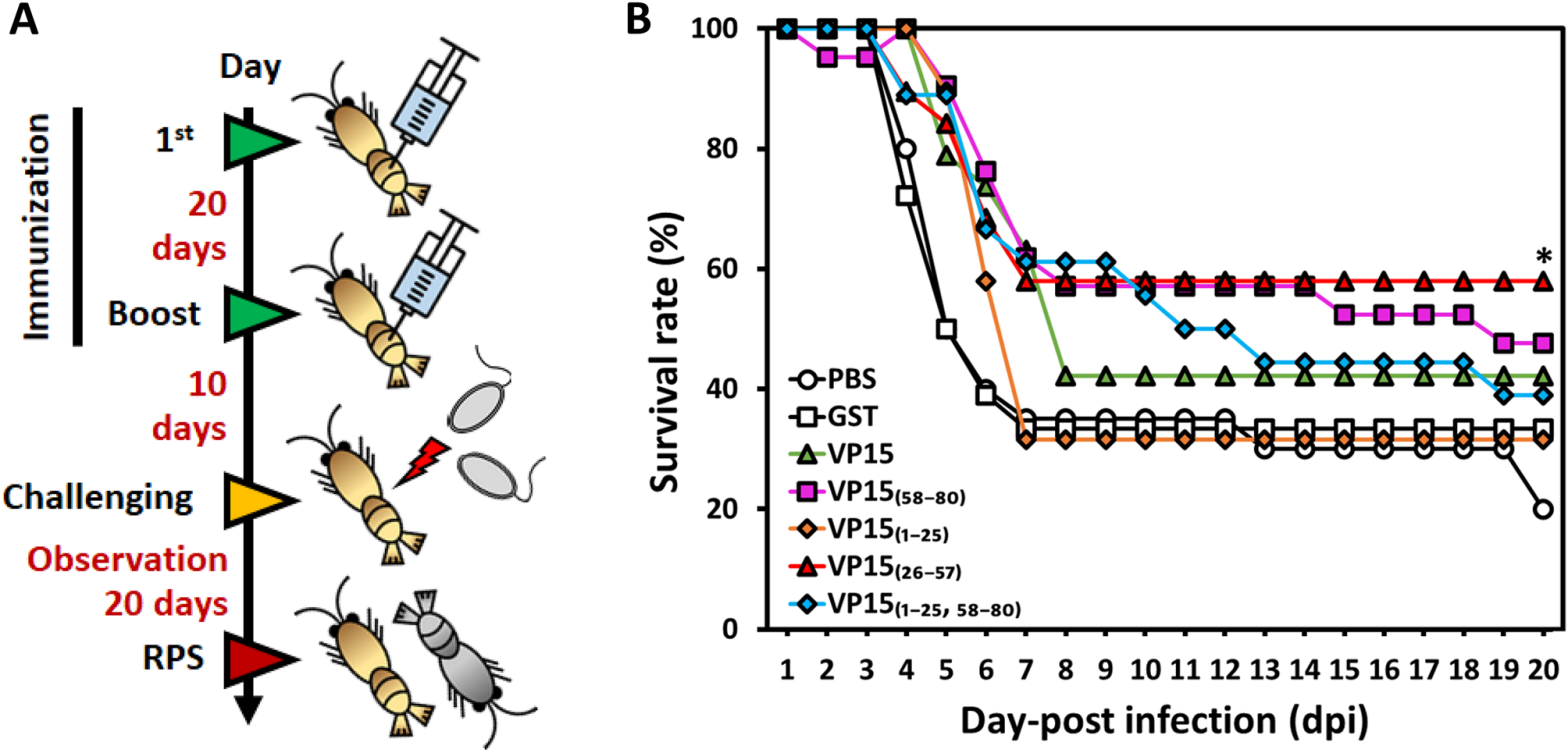
Evaluation of the *E. coli*-derived WSSV-VP15 and truncated VP15s for protective effects against WSSV. (**A**) Time schedule of shrimp immunization, WSSV challenge, and observation. The prime-and-boost immunization strategies for seven different groups (PBS, GST, VP15, VP15_(1–25)_, VP15_(26–57)_, VP15_(58–80)_, and VP15_(1–25, 58–80)_) are shown. In brief, shrimp were immune primed and boosted at day 0 and day 20 and challenged with WSSV via intramuscular injection 10 days afterward. (**B**) Time course of the survival rate of Kuruma shrimp after WSSV challenge. The mortality of shrimp was observed for 20 days at 24 h intervals. The line marked with an asterisk is significantly higher than that of the control groups.

The PBS and GST control groups showed a drastic decrease in survival rate from day 4 to day 7 and reached 20% and 33.3% (16% relative percent survival, RPS) at 20 days post infection (dpi). A group of shrimps receiving VP15_(1–25)_ showed a final survival rate of 31.6% (15% RPS), a similar trend to that of the GST group, indicating that VP15_(1–25)_ failed to provide a protective effect against WSSV. Groups of shrimp injected with VP15, VP15_(58–80)_, or VP15_(1–25,58–80)_ showed an survival rate at 20 dpi of 42.1% (28% RPS), 47.6% (35% RPS), and 38.9% (24% RPS), respectively. However, VP15_(26–57)_ provided a protective effect against WSSV with 57.9% of the survival rate (48% RPS) at 20 dpi (Fig. 2B, Table 1), which was the highest value among the experimental groups and was the only group showing a significant difference in survival rates compared with the controls.

**Table 1:** Mortality and RPS of VP15, truncated VP15 and SR11.

Treatment (prime-and-boost)	Number of dead individuals	Mortality (%)	RPS (%)
**Experiment 1**			
PBS (control)	16/20	80^a^	–
GST	12/18	67	16
VP15	11/19	58	28
VP15_(1–25)_	13/19	68	15
VP15_(26–57)_	8/19	42^b^	48
VP15_(58–80)_	11/21	52	35
VP15_(1–25,58–80)_	11/18	61	24
**Experiment 2**			
PBS (control)	7/15	47^a^	–
VP15_(26–57)_	2/11	18^b^	62
KR11	6/13	46	2
SR11	2/11	18^b^	62
SK10	5/11	46	2
KK13	7/13	46	2
**Experiment 3**			
PBS (control)	9/18	50^a^	–
VP15_(26–57)_	5/22	23^b^	54
SR11	6/22	27^b^	46

^a,b^*p* < 0.05.

### Vaccination and challenge experiment using the VP15-derived peptides

**Figure 3:**
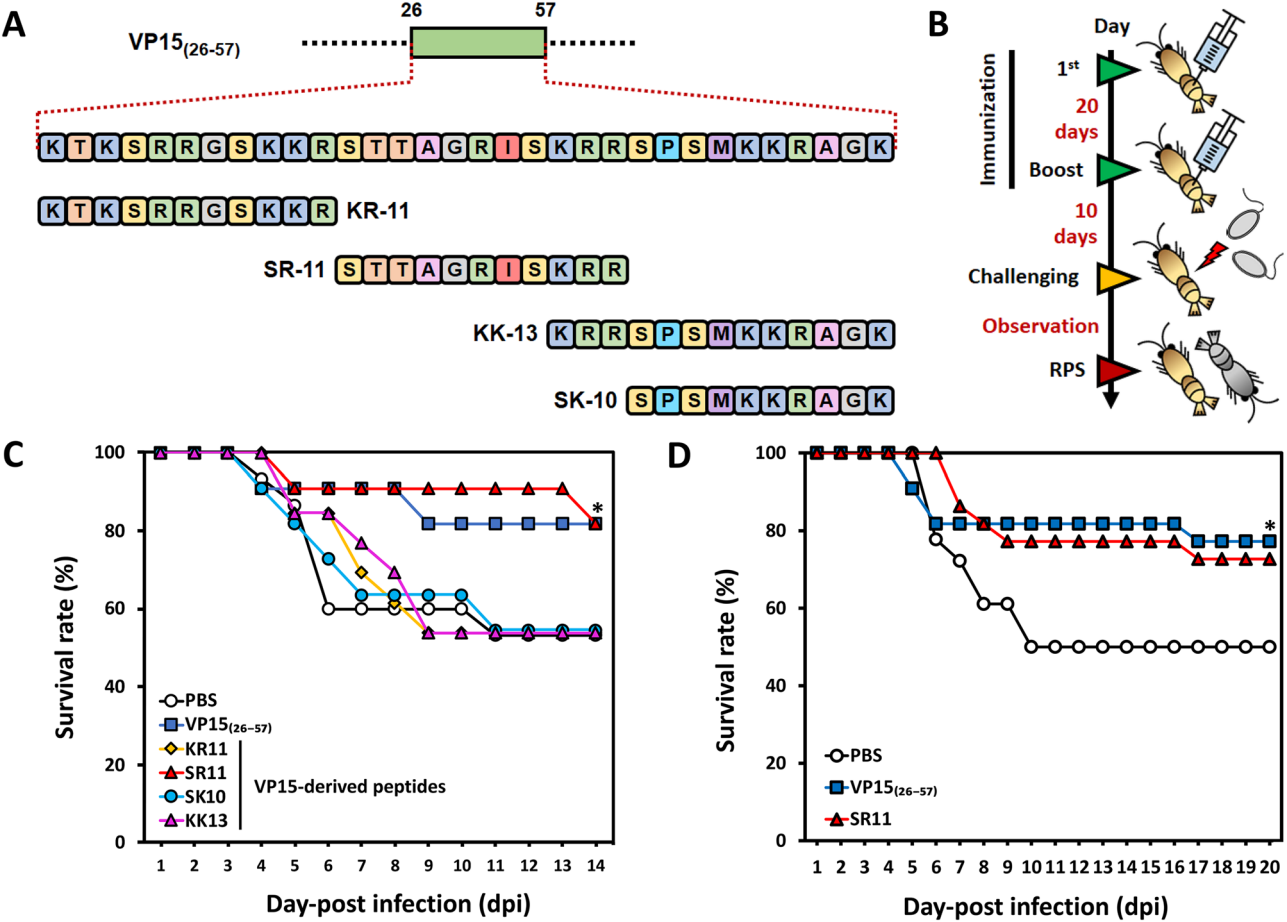
Peptide design and evaluation of VP15-derived peptides in shrimp against WSSV. (**A**) De novo design of peptides using the VP15_(26–57)_ amino acid sequence as a template. Four peptides, named KR11 (KTKSRRGSKKR), SR11 (STTAGRISKRR), SK10 (SPSMKKRAGK), and KK13 (KRRSPSMKKRAGK) were designed and chemically synthesized. (**B**) Time schedule of shrimp experiments using VP15-derived peptides as a protective agent. (**C**) Screening for the effective peptide by shrimp WSSV challenge experiment (KR11 (n = 13), SR11 (n = 11), SK10 (n = 11), or KK13 (n = 13)). Shrimp mortality was observed for 14 days, and the survival rate of each group was calculated. (**D**) Evaluation of SR11, an effective peptide, for enhancing shrimp survivability against WSSV. The experiment was conducted with twice the number of individuals (n = 25). For both experiments, VP15 _(26–57)_ was used as a positive control, while PBS served as a negative control.

To investigate the antigenic effects of VP15 at the peptide level, VP-15-derived peptides were designed based on the amino acid sequence of VP15_(26–57)_ and chemically synthesized (purity > 95%). As demonstrated in Fig. 3A, the peptides were KR11 (KTKSRRGSKKR), SR11 (STTAGRISKRR), SK10 (SPSMKKRAGK), and KK13 (KRRSPSMKKRAGK). Each peptide was IM-injected into the shrimp to screen for its protective effects against WSSV. Six groups of shrimp were set up with one PBS control group and five experimental groups. The protective effects of the four peptides were compared with those from GST-VP15_26–57_. The shrimp were immunized twice, as shown in Fig. 3B, with a dose of 10 µg/g shrimp of peptide or the recombinant protein and observed for 14 days after the WSSV challenge. Our screening experiment revealed that SR11 could provide a prominent protective effect with a final survival rate of ~ 80% (62% RPS), similar to GST-VP15_26–57_ at 14 dpi (62% RPS). The KR11-, SK10-, and KK13-injected groups showed a survival rate of 54% (2% RPS) at the end of observation, similar to the PBS group (Fig. 3C, Table 1). From this result, we interpreted that the KR11, SK10, and KK13 did not provide protective effects, as the survival rate was the same as that of the PBS control, while SR11 enhanced the resistance against WSSV. Therefore, SR11 was considered to be an antigenic peptide against WSSV in *M. japonicus*.

To verify the effect of the SR11 peptide, a larger sample size of 25 individuals was tested along with the positive control, GST-VP15_(26–57)_, and the PBS negative control group. The two experimental groups were treated similarly as Fig. 3B with the same dose of SR11 or GST-VP15_(26–57)_. As shown in Fig. 3D, shrimp rapidly died from day 6 to day 10 in the PBS group, with a final survival rate of ~ 50%. However, shrimp in the group receiving either GST-VP15_(26–57)_ or SR11 showed survival rates of 77% (54% RPS) and 73% (46% RPS), respectively, at 20 dpi (Table 1), indicating that SR11 could generate reproducible anti-WSSV effects.

### Sequence analysis, alignment, and phylogenetic analysis of MjgC1qR

The gC1qR of invertebrates has been suggested to be involved in innate immunity against pathogens by acting as a pathogen recognition receptor^43,44^. There is evidence showing that gC1qR might act as a PRR to recognize a broad range of non-self-components such as bacteria, virus, and PAMPs and can be upregulated upon WSSV infection^45,46^. We hypothesized that gC1qR from *M. japonicus* (MjgC1qR) might also interact with VP15. gC1qRs have been identified from many invertebrate species but not from Kuruma shrimp. We then attempted to generate and analyze the cDNA sequence of MjgC1qR. To this end, bioinformatic analysis was performed to compare MjgC1qR with the reported gC1qR(s) from the database. The obtained full-length cDNA of MjgC1qR was 1120 bp, consisting of 98 bp of a 5’-untranslated region (UTR), 786 bp of an open reading frame (ORF), and 336 bp of a 3’-UTR (including the stop codon) with one mRNA instability element (ATTTA). The ORF encodes a 262 amino acid-long protein with an MW of 29.2 kDa and a theoretical *pI* of 4.74. The protein also contains an N-terminal mitochondrial targeting sequence with a length of 44 amino acids, a mitochondrial acidic matrix (MAM33) domain (residues 78–257) at its C-terminus, and one arginine-glycine-aspartic acid (RGD) motif (Fig. S5 of Supplementary information). Comparative multiple alignment of gC1qR amino acid sequences from various species revealed a highly conserved MAM33 domain and cell attachment RGD motif across the invertebrate species. MjgC1qR also shared high identity with other invertebrate gC1qRs (93.5% identity with *Penaeus vannamei*, 92% with *Penaeus monodon*, 90.5% with *Penaeus chinensis*, 74.3% with *Eriocheir sinensis*, 71.6% with *Portunus trituberculatus*, 71% with *Macrobrachium rosenbergii*, 70.2% with *Palaemon carinicauda*, 69.8% with *Macrobrachium nipponense*, and 68.7% with *Pacifastacus leniusculus*) (Fig. S6 of Supplementary information). The phylogenetic tree revealed that MjgC1qR had a close evolutionary relationship to crustacean gC1qRs, especially gC1qR of *P. vannamei* (Fig. S7 of Supplementary information).

### Expression of MjgC1qR using the silkworm-bacmid expression vector system

**Figure 4:**
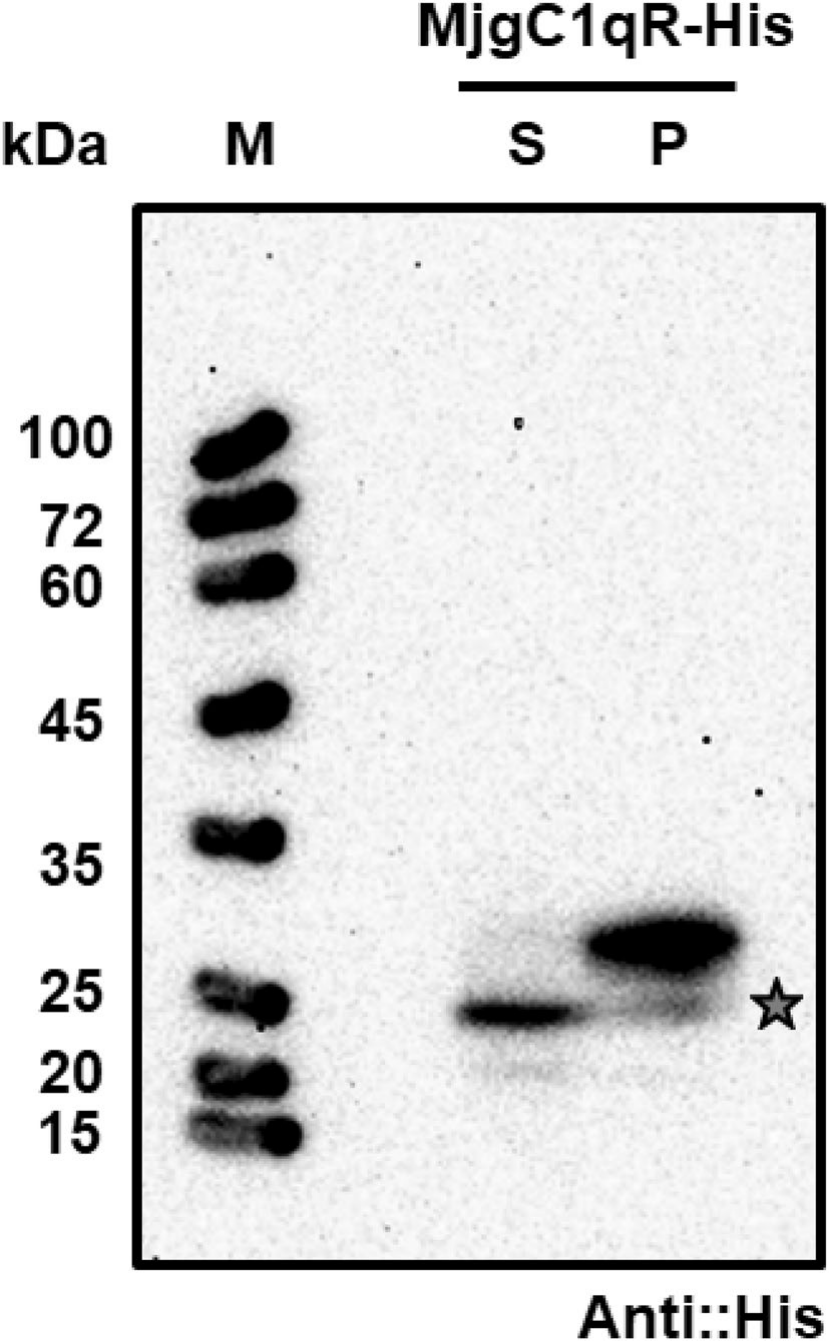
Western blot analysis of MjgC1qR expressed in silkworm larvae. Western blot analysis of silkworms was performed with an anti-His antibody. Silkworms were transfected with recombinant bacmid harboring the MjgC1qR-His construct, and fat bodies were collected after 5 days of transfection. M: marker; S: supernatant; and P: precipitate.

The bacmid harboring the full-length MjgC1qR-His gene was directly injected for transfection of silkworm larvae. Hemolymph and fat body were collected at 5 dpi. Fat bodies were homogenized and analyzed for the expressed protein via Western blot analysis. As shown in Fig. 4 (Fig. S8 of Supplementary information**)**, MjgC1qR was expressed as a single protein product in silkworm larvae under the control of the polyhedrin promoter. The protein was detected in the silkworm fat body in both a soluble and an aggregated form by the anti-His antibody. The size of the soluble MjgC1qR was found to be smaller than that of the aggregated form, with lengths of approximately 25 and 29 kDa, respectively, possibly due to the cellular processing of the signal peptide.

### VP15 and the SR11 peptide interact with MjgC1qR

**Figure 5:**
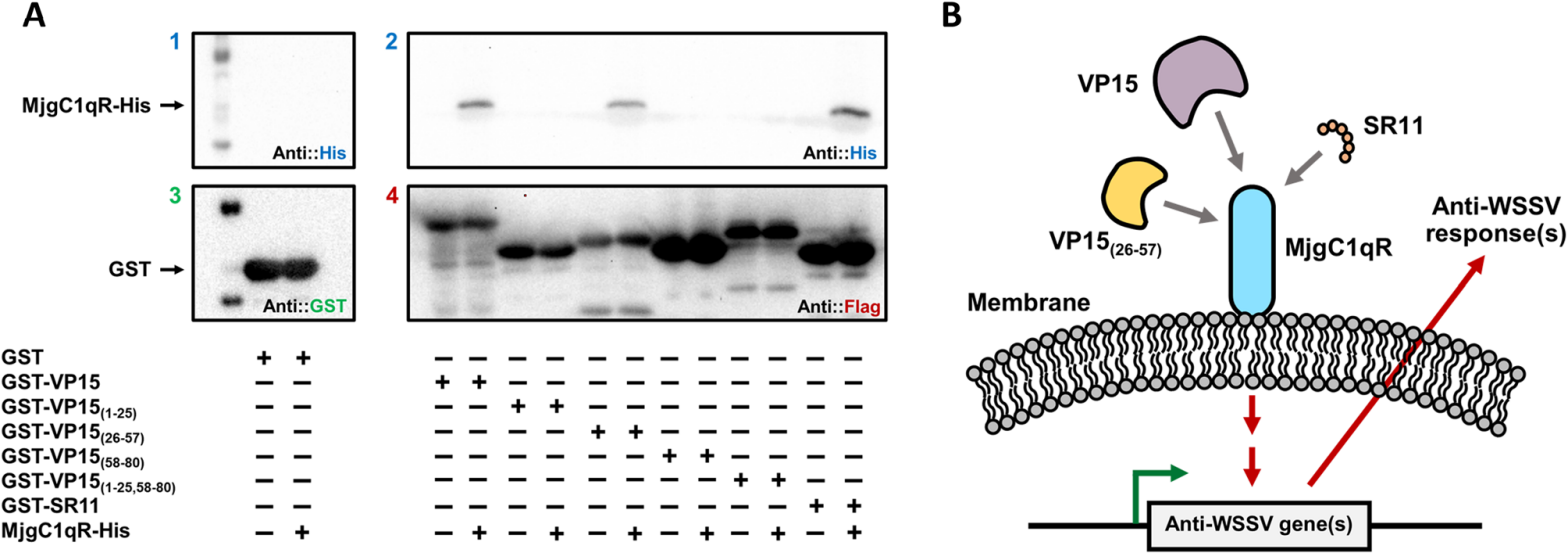
GST pull-down assay between MjgC1qR and GST-VP15, GST-VP15_(1–25)_, GST-VP15_(26–57)_, GST-VP15_(58–80)_, GST-VP15_(1–25,58–80)_, GST-SR11, and a proposed antiviral mechanism. (**A**) The interactions of MjgC1qR-His with GST-VP15, GST-VP15_(1–25)_, GST-VP15_(26–57)_, GST-VP15_(58–80)_, GST-VP15_(1–25,58–80)_, GST-SR11 were observed via GST pull-down assay and confirmed by Western blot analysis with (1 and 2) anti-His antibody, (3) anti-GST antibody, and (4) anti-Flag antibody. The symbol “ + ” indicates the proteins used for the assay. (**B**) In shrimp, VP15, VP15_(26–57)_, and SR11 interacted with the host gC1qR, hence inducing a cellular signaling cassette that could activate the transcription of antimicrobial peptides.

The interactions of MjgC1qR with the candidate bait proteins were observed through GST pull-down assay. In this study, the candidate baits were GST-VP15, GST-VP15_(26–57)_, and the GST-fused peptide GST-SR11, as these proteins and peptides could provide protective effects in shrimp against WSSV. Before this experiment, GST-SR11 was expressed in *E. coli* and purified (Figs. S9, S10 of Supplementary information)). Additionally, pull down assays between MjgC1qR and GST-VP15_(1–25)_, GST-VP15_(58–80)_, or GST-VP15_(1–25,58–80)_ were performed. As shown in Fig. 5A (Figs. S11–S14 of Supplementary information), the recombinant MjgC1qR protein interacted with VP15, VP15_(26–57)_, and the SR11 but not with the control GST protein and the other tested proteins.

## Discussion

VP15 of WSSV has been identified as one of the major WSSV structural proteins in the nucleocapsid fraction and has an affinity for nucleic acids, especially supercoiled DNA^41^. Our aim was to identify an antigenic determinant of VP15 that could enhance the shrimp survival rate upon WSSV infection. We designed truncated constructs by dividing VP15 into three regions: the N-region (first 25 amino acids), middle region (32 amino acids), and C-region (last 23 amino acids), and purified truncations from *E. coli* were then tested as “vaccines” in shrimp. Purification of GST-VP15 suggested that the full-length protein might not be a stable protein, as it tended to be fragmented, as confirmed by SDS-PAGE analysis (Fig. 1E). The full-length VP15 protein (80 aa) has a theoretical size of approximately 35 kDa and is rich in serine (20%), arginine (20%), and lysine (21.2%). After purification, we observed protein bands below 35 kDa and a thick protein band at > 30 kDa, which could not be observed by Western blotting using anti-Flag antibody. Moreover, the truncated VP15s could be purified with a main band corresponding to the predicted MW along with minor bands below the main products, except for VP15_(1–25,58–80),_ which resulted in several productive bands below the main product similar to the Western blot of purified proteins using anti-GST antibody (Figs. 1E, S3 of Supplementary information).

To our knowledge, VP15 has barely been used as a protective agent against WSSV in penaeid shrimp, as this protein is usually not considered a part of the “infectome,” a protein complex that comprises 14 different proteins, including VP19, VP24, VP26, and VP28, located on the viral surface^21^. VP15 was applied as a DNA vaccine in *Peneus monodon*, but it could not elicit any protection against WSSV^42^*.* In our previous study, we found that VP15, when administered to Kuruma shrimp IM at a dose of 0.04 mg/g shrimp using the prime-and-boost method, increased the survival rate to 78% at 20 dpi. Moreover, the same GST-VP15 revealed a different result, in which the final survival rate was approximately 43% with a dose of 0.01 mg/g shrimp using the same protocol^38^. This difference can be explained by the amount of the recombinant protein delivered to the shrimp. The dose was four times lower than the previous one. Therefore, we assumed that the protective effect of VP15 was dose dependent.

Hence, in this study, the antigenic site of VP15 was determined via in vivo vaccination studies in Kuruma shrimp using an *E. coli*-derived VP15-truncated series including VP15_(1–25)_, VP15_(26–57)_, VP15_(58–80)_, and VP15_(1–25,58–80)_. Remarkably, only the group receiving VP15_(26–57)_ showed a promising survival rate higher than that obtained with VP15 and was significantly different from the controls. Based on the results, we hypothesized that VP15_(26–57)_ might contain a specific region responsible for the anti-WSSV activity. To identify whether there is an antigenic determinant on VP15_(26–57)_, the amino acid sequence of VP15_(26–57)_ (KTKSRRGSKKRSTTAGRISKRRSPSMKKRAGK) was taken into account. Among the designed KR11, SR11, KK13, and SK10 peptides, only the SR11 group showed an improved survival rate at the end of observation, and this was reconfirmed in a larger sample size group. The results were in good agreement with those of the previous experiment (Fig. 3C,D). Both experiments were performed in comparison to the parental protein VP15_(26–57)_. Both shrimp receiving VP15_(26–57)_ and those receiving SR11 showed a very similar trend during the observation periods, indicating that SR11 could be an antiviral determinant of VP15_(26–57)_ responsible for triggering protective effects in vivo.

gC1qR is considered a receptor for the globular head of C1q of the classical complement pathway^47,48^. gC1qR has been found on a cell surface acting as a receptor for a broad range of proteins and as a pathogen recognition receptor of innate immunity in many invertebrate species, suggesting that the protein may play a crucial role in an innate immune response against pathogenic bacteria and viruses^44,45,49^. We hypothesized that MjgC1qR might interact with VP15 as well. Therefore, a novel MjgC1qR was identified from *M. japonicus* and expressed with the His-tag in the silkworm-bacmid expression system. Mammalian gC1qRs and reported crustacean gC1qRs contain mitochondrial targeting sequences at the N-terminus and MAM33 domains at the C-terminus, which direct the protein to the mitochondrial matrix for cleavage and maturation^43,45,50–52^. We also confirmed in the current study that MjgC1qR also has similar domains, indicating that MjgC1qR belongs to the gC1qR family and might function in the same way. Here, silkworm expression of recombinant MjgC1qR yielded a similar result to a previous report in which recombinant mammalian gC1qR expressed in Sf9 cells was processed due to the mitochondrial targeting sequence^52^. As investigated in GST pull-down assays, VP15, VP15_(26–57),_ and SR11 interacted with MjgC1qR but not with the other truncated constructs (Fig. 5A).

As illustrated in Fig. 5B, the current study provides a possible clue that the antiviral immune response could be triggered through an interaction between MjgC1qR and GST-VP15, GST-VP15_(26–57)_, and GST-SR11. Based on the in vivo experiment and in vitro GST pull-down assay, it can be concluded that SR11 might be a linear antiviral determinant or an epitope of VP15. It can be explained that only a group of shrimps receiving SR11 showed an improvement in the survival rate. It is conceivable that shrimp might recognize “STTAGRISKRR” of VP15 as a nonself component through the pattern recognition receptors of their innate immune system, such as gC1qR, hence enhancing their response against the injected antigen.

Recently, “trained immunity” or “immune priming” has been evidenced in invertebrate innate immune systems, such as those of shrimp^31,53^. Immune priming in shellfish has been extensively studied as the priming effects could effectively induce immune responses by administering specific antigens or killed/deactivated virus either septic or oral routes. Several studies have demonstrated that shrimp immune priming with inactivated pathogenic bacteria (*e.g., Vibrio harveyi*, *V. alginolyticus,* and *V. anguillarum*) could protect them from vibriosis^54,55^. In addition, evidence has indicated an improved survival rate in shrimp primed with killed or attenuated WSSV^31,56^ or with WSSV structural subunits. Thus far, the priming effects can be evaluated by specific parameters from humoral and cellular responses as well as survival rate, the efficiency of pathogen clearance, rate of phagocytosis, expression of immune-related genes, etc.^24^. Immune priming has been found to promote phagocytosis, encapsulation, apoptosis, clotting, and nodulation levels of cellular responses and humoral responses by an increase in production of various molecules, e.g., phenoloxidase, AMPs, lysozyme, and lectins in primed animals^26–30^. The generation of immune-related molecules may require a specific recognition between receptors and their ligands through the key-lock principle^57^. In here, we purposed the interaction of VP15 to gC1qR of *M. japonicus* might induce an immune response hence, resulting in an improvement of shrimp survivability after exposure to WSSV. gC1qR is known to inhibit RIG-I-like receptor (RLR) dependent NF-κB signaling of mitochondrial antiviral signaling (MAVS) pathways by interacting with the MAVS protein^58^. Recently, MAVS-related signaling molecules have been identified from invertebrate species^59^; however, the study is limited. The interaction of VP15 to MjgC1qR might positively affect the MAVS pathway by interfering with the inhibitory effect of gC1qR to MAVS protein.

In conclusion, the antigenicity and immunogenicity of recombinant truncated VP15s and VP15-derived peptides were identified via in vivo animal experiments in Kuruma shrimp (*M. japonicus*) with a prime-and-boost strategy. SR11 (STTAGRISKRR) derived from VP15_(26–57)_ was successfully identified as an anti-WSSV agent, showing an effective peptide to enhance shrimp persistence against WSSV.

## Materials and methods

### Construction of truncated WSSV-VP15 for expression in *E. coli*

In a previous study, the WSSV-VP15 gene (*wsv214*) fused with a C-terminal Flag-tag (DYKDDDDK) was successfully cloned into pGEX-6P-1, named ‘pGEX-VP15′^38^. For the construction of a series of truncated VP15 proteins (Fig. 1A), inverse PCR was performed with KOD-PLUS-NEO (Toyobo, Japan) using pGEX-VP15 as a template. The primers are listed in Table 2. The amplicons were then treated with T4 DNA polymerase (NEB, Tokyo, Japan) according to the manual, self-ligated, and transformed into competent *E. coli* DH5α by the heat-shock method. Colony PCRs were performed using pGEX-FW and pGEX-RV for a comparison between a full-length VP15 and the truncated VP15 series. The pGEX harboring each truncated VP15 gene was prepared from the positive clones, and DNA sequences were verified by Sanger DNA sequencing. For the construction of pGEX-SR11 with a C-terminal Flag-tag, pGEX-VP15 was used as a template in inverse PCR with PrimeSTAR MAX DNA polymerase (Takara, Japan) using pGEX-InvFW-ISKRR-FLAG and pGEX-InvRV-STTAGR (Table 2). The amplicon was then self-ligated, propagated in *E. coli* DH5α, and verified via DNA sequencing.

**Table 2:** Primers used in this study.

Name	Sequence (Direction from 5′ to 3′)
pGEX-FW	GAAGTTCTGTTCCAGGGGCCC
pGEX-RV	AGGCAGATCGTCAGTCAGTCA
pFastBac-FW	TATTCCGGATTATTCATACC
pFastBac-RV	ACAAATGTGGTATGGCTGATT
pET41-RV	GGTTATGCTAGTTATTGCTC
**VP15**_**(1–25)**_	
pGEX-InvFW-Flag	GACTACAAGGATGACGATGACAAGTAAG
VP15tr(aa1–25)-RV	GGAGGAGCGAGCCACCATCTTCAG
**VP15**_**(58–80)**_	
VP15tr(aa58–80)-FW	AAGTCCTCCACCGTGCGT
pGEX-InvRV-ATG	CATGGATCCCAGGGGC
**VP15**_**(1–25, 58–80)**_	
VP15tr(aa58–80)-FW	AAGTCCTCCACCGTGCGT
VP15tr(aa1–25)-RV	GGAGGAGCGAGCCACCATCTTCAG
**VP15**_**(26–57)**_	
pGEX-InvFW-Flag	GACTACAAGGATGACGATGACAAGTAAG
VP15tr(aa26–57)-RV	TTTGCCAGCGCGCTTCTT
**VP15**_**(SR–11)**_	
pGEX-InvFW-ISKRR-FLAG	ATCTCCAAGCGTCGTGACTACAAGGATGACGATGACAAG
pGEX-InvRV-STTAGR	GCGGCCAGCGGTGGTGGACATGGATCCCAGGGGCCCCT
**MjgC1qR**	
MjgC1qR-FW-BamHI	ACCAGGATCCATGAGTGCCATCAGTCGTGC
MjgC1qR-RV-XhoI-NoStop	TTGTCTCGAGTTTCCTCTTGACAAAGTCCT

### Expression and purification of recombinant proteins from *E. coli*

The verified plasmids were electroporated into *E. coli* Rosetta gami-B (Novagen, Inc., Tokyo, Japan) before expression of the recombinant protein. Transformed *E. coli* Rosetta gami-B cells were grown overnight at 37 °C in Lurie-Bertani (LB) broth supplemented with 50 µg/ml ampicillin (LB + Amp). The inoculums were then transferred to baffled flasks containing 250 ml of LB + Amp medium and incubated at 37 °C with shaking at 150 rpm. When the OD_600_ reached 0.5, the culture was cooled on ice for 30 min, protein expression was induced by adding isopropyl β-D-1-thiogalactopyranoside (IPTG) to a final concentration of 0.5 mM in the culture, and incubation was continued for 18 h at 16 °C. After 18 h, the cells were collected by centrifugation (6,000 × g, 4 °C, 15 min), washed twice with phosphate-buffered saline (PBS, pH 7.3), and stored at − 80 °C until use. Protein expression was analyzed via sodium dodecyl sulfate–polyacrylamide gel electrophoresis (SDS-PAGE) and Western blotting using anti-Flag antibody (1:10,000, MBL, Japan).

Before protein purification, the cells were resuspended in PBS containing 1 × proteinase inhibitor (cOmplete Mini, EDTA-free Protease Inhibitor Cocktail, Sigma-Aldrich, Tokyo, Japan) and 10 µg/ml lysozyme. The suspension was sonicated on ice (70% amplitude, 30 s on/off, 15 cycles), centrifuged (10,000 × g, 4 °C, 10 min), and filtered through a 0.2 µm cellulose acetate membrane (Minisart NML, Sartorius, Tokyo, Japan). The GST-fused recombinant proteins were purified by GST affinity chromatography (Glutathione Sepharose 4 Fast Flow, GE Healthcare, Tokyo, Japan). Protein concentrations were determined by a Pierce BCA Protein Assay Kit (Thermo Fisher Scientific, Tokyo, Japan) after dialysis against PBS using an Amicon Ultra-15 30 K Centrifugal Filter Unit (Merck Japan, Tokyo, Japan).

### Cloning of the full-length MjgC1qR gene

Total RNA was extracted from adult Kuruma shrimp (10.1 cm of total length and 8.1 g of body weight) using ISOGEN (Nippon Gene, Tokyo, Japan) according to the manufacturer’s protocol. First-strand cDNA was synthesized with 5 μg of total RNA using ReverTra Ace (Toyobo, Shiga, Japan) and was kept at − 30 °C until use. The obtained cDNA was used as a template for amplifying the MjgC1qR gene without a stop codon using MjgC1qR-FW-BamHI and MjgC1qR-RV-XhoI-NoStop. The final PCR products were cloned into pET-41a( +) (MERCK Japan) to fuse the gene with a polyhistidine-tagged sequence at the C-terminus (designated pET41-MjgC1qR-His), and positive clones were selected and sequenced. MjgC1qR-His was amplified from pET41-MjgC1qR-His using MjgC1qR-FW-BamHI and pET41-RV and cloned into pFastbac-1 (Thermo Fisher Scientific K. K, Tokyo, Japan), and the product was named pFB-MjgC1qR-His. The sequence was confirmed again via DNA sequencing. All the primers (including their sequences) are listed in Table 2.

### Sequence analysis of the MjgC1qR gene

The ORF finder (https://www.ncbi.nlm.nih.gov/orffinder/) was used to predict the amino acid sequence. The predicted sequence was analyzed by BLAST (https://blast.ncbi.nlm.nih.gov/Blast.cgi). The protein mass and isoelectric point were theoretically determined by computing the pI/Mw tool (https://web.expasy.org/compute_pi/). Mitochondrial targeting sequences and protein domain features were predicted using SMART (Simple Modular Architecture Research Tool, http://smart.embl-heidelberg.de/)^60^ and MITOPROT (https://ihg.gsf.de/ihg/mitoprot.html)^61^, respectively. Multiple alignment analysis of MjgC1qR was performed via Clustal Omega software (https://www.ebi.ac.uk/Tools/msa/clustalo/). A phylogenetic tree of gC1qR(s) was generated using the neighbor-joining method in MEGA 7.0.

### Generation of a recombinant bacmid encoding MjgC1qR-His and protein expression in silkworm (*Bombyx mori*) larvae

The positive recombinant plasmid pFB-MjgC1qR-His was used to transform *E. coli* BmDH10Bac for the generation of a recombinant *Bombyx mori* nucleopolyhedrovirus (BmNPV) bacmid^62^. The bacmid was then transfected into silkworm larvae. Silkworm larvae (Ehime Sansyu, Ehime, Japan) were reared for 5 days with an artificial diet, Silkmate S2 (Nosan, Japan), under a controlled environment (25 °C, 65 ± 5% relative humidity). Silkworm hemolymph and fat bodies were collected 5 days after bacmid injection. Hemolymph was kept at − 80 °C as a BmNPV stock for protein expression in silkworms. The fat body was resuspended in lysis buffer (20 mM Tris–HCl, 140 mM NaCl, and 0.1% Triton X-100, pH 7.6) with proteinase inhibitor added, sonicated, centrifuged, and clarified through a 0.45 μm filter. The clarified lysate was subjected to protein expression analysis by Western blotting with anti-His antibody (MBL, Nagoya, Japan).

### GST pull-down assays

The GST pull-down assay was modified based on a method published by Nguyen and Goodrich^63^. In the assay, glutathione Sepharose 4B resins (GE Healthcare Japan, Tokyo, Japan) were first washed and equilibrated four times with PBS. The bait proteins GST (as a negative control), GST-VP15, GST-VP15_(1–25)_, GST-VP15_(26–57)_, GST-VP15_(58–80)_, GST-VP15_(1–25,58–80)_ or GST-SR11 were immobilized on the resins by adding 150 μg of each purified recombinant product and incubated for 4 h at 4 °C on a rotator shaker. After removing the supernatant, the resins were washed two times with ice-cold TGEM (1.0) [20 mM Tris–HCl (pH 7.9), 20% glycerol, 5 mM MgCl_2_, 0.1% NP-40, 0.2 mM phenylmethylsulfonyl fluoride (PMSF) and 1.0 M NaCl] and two times with ice-cold TGMC (0.1) [20 mM Tris–HCl (pH 7.9), 20% glycerol, 5 mM MgCl_2_, 5 mM CaCl_2_, 0.1% NP-40, 0.2 mM PMSF and 0.1 M NaCl]. Then, silkworm extract containing MjgC1qR-His was dialyzed into TGMC (0.1) with 0.1% Triton X-100. MjgC1qR-His was then added to the immobilized proteins, and the mixture was incubated overnight at 4 °C on a rotator shaker. After incubation, the resins were washed four times with ice-cold TGEM (0.1) [20 mM Tris–HCl (pH 7.9), 20% glycerol, 5 mM MgCl_2_, 0.1% NP-40, 0.2 mM phenylmethylsulfonyl fluoride (PMSF) and 0.1 M NaCl] to remove the unbound target protein. The immobilized proteins on the resins were analyzed by Western blotting against GST-tag (anti-GST-tag mAb, MBL, Japan), Flag-tag, and His-tag.

### Synthesis of the peptides

Four VP15-derived peptides (KR11, SR11, SK10, and KK13) (Fig. 4A) were commercially synthesized (GL Biochem Ltd., Shanghai, China). The peptide characteristics were analyzed via high-performance liquid chromatography (HPLC) and electrospray ionization mass spectrometry (ESI–MS). HPLC was employed for purification of each peptide using an Inertsil ODS-SP column (purity > 95%). The purity and molecular masses of purified synthetic peptides were analyzed using electrospray ionization coupled with liquid chromatography-mass spectrometry (LC–MS/ESI, Agilent-6125B).

### Shrimp and WSSV inoculum

Kuruma shrimp (*Marsupenaeus japonicus*) and the WSSV inoculum were prepared according to a previous method^64^. In brief, shrimp with a mean body weight (MBW) of 3.1 to 6.8 g were maintained in dechlorinated electrolyzed seawater (33.05 ± 0.13 parts per trillion) at 24 ± 1.8 °C using double-bottomed tanks with sand beds and were fed a commercial diet (shrimp feed, Juveniles P-2, Maruha, Tokyo, Japan) at 3% of their body weight per day.

Adult *M. japonicus* (MBW 78 g) were intramuscularly (IM)-inoculated with a 10^–3^ dilution of a virus prepared from naturally WSSV-infected juvenile shrimp. The hemolymph was withdrawn after 3 days of infection and stored at − 80 °C. Before each experiment, an aliquot of the stored virus was thawed and centrifuged at 1500 × g at 4 °C for 10 min. The resultant supernatant was diluted in PBS to 10^–4.8^ for the challenge test.

### Vaccination and intramuscular (IM) challenge experiment

#### Experiment 1: protective effect of truncated VP15s

Kuruma shrimp (MBW 3.16 g, n = 18–21) were divided into seven groups: five experimental groups and two mock treatment groups. Each group comprised 20 individuals. Shrimp in each experimental group were (1st-injection) IM-vaccinated with VP15, VP15_(1–25)_, VP15_(26–57)_, VP15_(58–80)_, or VP15_(1–25,58–80)_ at a dose of 10 μg/g shrimp. The shrimp were (2nd-injection) immune-boosted again at 20-day intervals and then IM-challenged with WSSV at a dose of 2.69 × 10^4^ DNA copies/shrimp 10 days after boosting (Fig. 2A). In the mock treatment groups, shrimp were injected with either PBS or GST under the same procedures as in the experimental groups. Shrimp mortality was observed for 20 d at 24 h intervals, and the relative percent survival (%, RPS) was calculated with the formula proposed by Amend^65^ as follows:

$$ RPS = \left\{ {1 - \left( {\frac{{{\text{\% }}\;{\text{mortality}}\;{\text{in}}\;{\text{immunized}}\;{\text{group}}}}{{{\text{\% }}\;{\text{mortality}}\;{\text{in}}\;{\text{PBS}}\hbox{-}{\text{injected}}{{\text{group}}}}}} \right)} \right\} \times 100. $$

#### Experiment 2: screening for an effective peptide derived from VP15

For screening of an effective peptide, juvenile Kuruma shrimp in each group were injected with KR11 (n = 11–13), SR11 (n = 11), SK10 (n = 11), or KK13 (n = 13) at a dose of 10 μg/g shrimp. Shrimps in each group were then again injected with the peptide to boost their immunity at 20-day intervals. A challenge experiment was performed 10 days after the second injection by injecting the virus at a dose of 2.69 × 10^4^ copies/shrimp, as mentioned above. Shrimps were observed for 15 days, and RPSs were calculated (Fig. 3B). The protective effect of the peptides was compared with that of VP15_(26–57)_ (n = 11).

#### Experiment 3: evaluation of SR11, the VP15-derived effective peptide

SR11 was found to be an effective peptide in *Experiment 2*. SR11 was then verified to have protective effects against a larger shrimp group than VP15_(26–57)_. Two experimental groups and one PBS control group with 18–22 individuals/group were set up. Shrimp were injected twice with 10 μg/g shrimp of the peptide or the recombinant protein at 20-day intervals. Ten days after a second injection, shrimp were challenged with WSSV (Fig. 3B). The mortality rate was observed for 20 days, and the RPS was calculated.

### Statistical analysis

Statistical analysis of the time-mortality relationship was performed with Kaplan–Meier analysis (χ^2^ test) at a 5% confidence level.

## Supplementary Information

Supplementary Information.
