# Supplementary material for: Identification of antigenic domains and peptides from VP15 of white spot syndrome virus and their antiviral effects in Marsupenaeus japonicus
Source: Sci Rep. 2021 Jun 17;11:12766. doi: 10.1038/s41598-021-92002-8 (PMC8211838; doi:10.1038/s41598-021-92002-8)
Supplement: Supplementary file 1 — Supplementary Information. [file 41598_2021_92002_MOESM1_ESM.pdf]

Supplementary Information

Identification of antigenic domains and peptides from VP15 of white spot syndrome virus and their antiviral effects in *Marsupenaeus japonicus*

Jirayu Boonyakida[1], Jian Xu[2], Jun Satoh[3], Takafumi Nakanishi[4], Tohru Mekata[5], Tatsuya Kato[1,4,6], and Enoch Y. Park[1,4,6,*]

✉ Enoch Y. Park
park.enoch@shizuoka.ac.jp

[1] Department of Bioscience, Graduate School of Science and Technology, Shizuoka University, 836 Ohya, Suruga-ku, Shizuoka 422-8529, Japan
[2] Institute of Biology and Information Science, Biomedical Synthetic Biology Research Center, School of Life Sciences, East China Normal University, Shanghai 200062, PR China
[3] Fisheries Technology Institute of National Research and Development Agency, Japan Fisheries Research and Education Agency, Tamaki Field Station, Mie 519-0423, Japan
[4] Department of Applied Biological Chemistry, Graduate School of Integrated Science and Technology, Shizuoka University, 836 Ohya, Suruga-ku, Shizuoka 422-8529, Japan
[5] Fisheries Technology Institute of National Research and Development Agency, Japan Fisheries Research and Education Agency, Namsei Field Station, Mie 516-0193, Japan
[6] Research Institute of Green Science and Technology, Shizuoka University, 836 Ohya, Suruga-ku, Shizuoka 422-8529, Japan

E-mail:
jirayu.boonyakida.17@shizuoka.ac.jp (JB)
xujian@sei.ecnu.edu.cn (JX)
junsatoh@affrc.go.jp (JS)
nakanishi19951203@gmail.com (TN)
mekata@affrc.go.jp (TM)
kato.tatsuya@shizuoka.ac.jp (TK)
park.enoch@shizuoka.ac.jp (EYP)

A

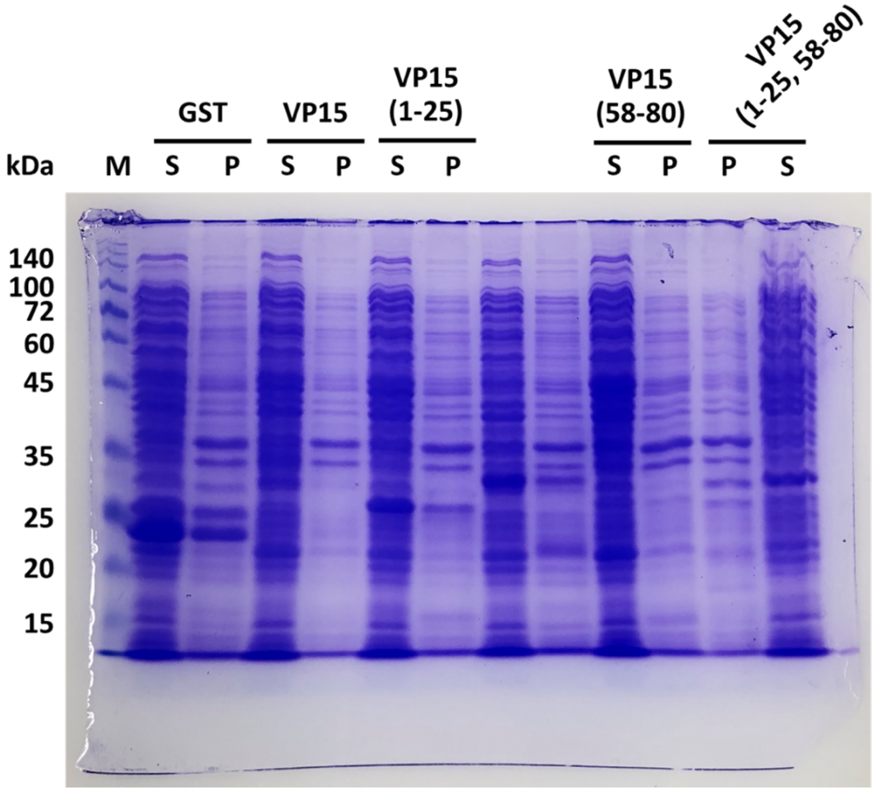
GST
VP15
VP15 (1-25)
VP15 (58-80)
VP15 (1-25, 58-80)
kDa
M
S P S P S P S P P S
140
100
72
60
45
35
25
20
15

B

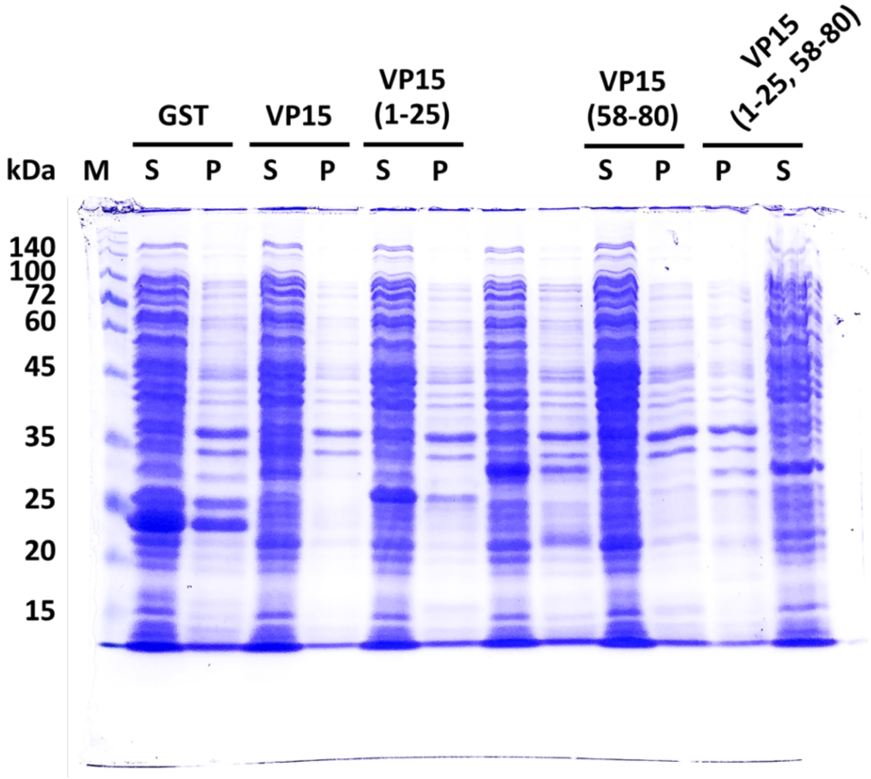
GST
VP15
VP15 (1-25)
VP15 (58-80)
VP15 (1-25, 58-80)
kDa
M
S P S P S P S P P S
140
100
72
60
45
35
25
20
15

C

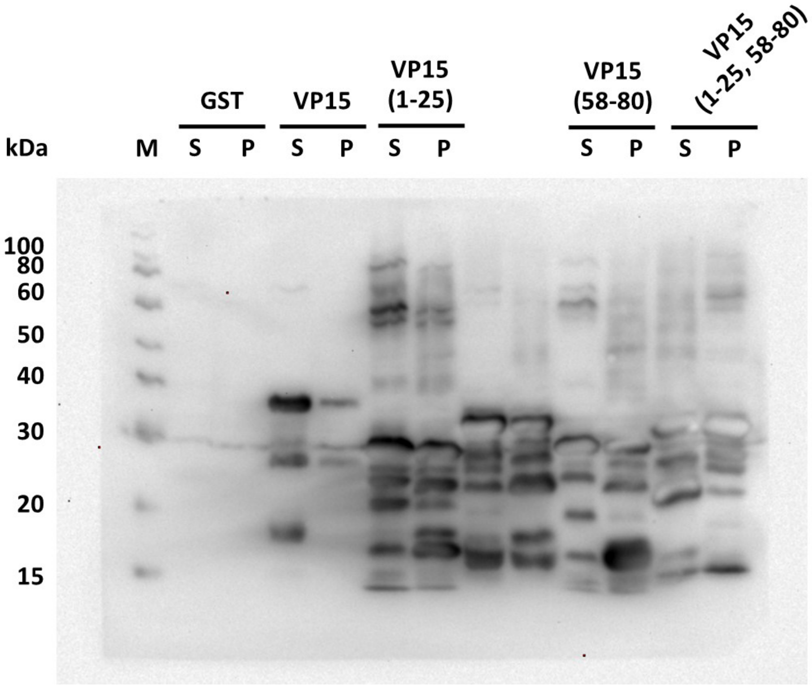

Figure S1. These are the original file (full-length gel and blot) for Figure 1C and 1D. Original photo without contrast (**A**) and exposure increased (**B)** in a CBB staining for the recombinant proteins expressed in *E. coli* (Figure 1C panel). Western blotting against the Flag-tag for verifying the target proteins for Figure 1D (**C**). The lanes without labeling are irrelevant results.

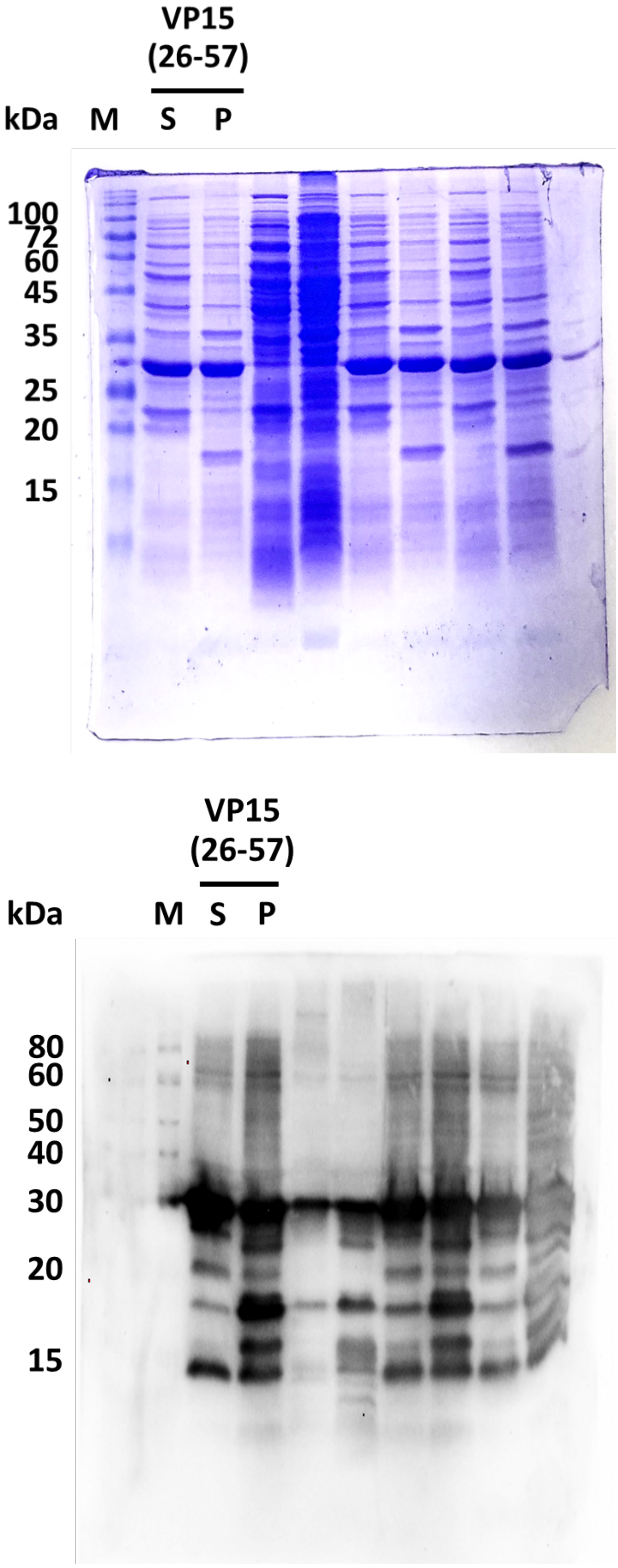

Figure S2. These are the original full-length gel and blot for Figure 1C and 1D of GST-fused VP15$_{(26-57)}$ expressed in *E. coli* cells. Upper panel is a CBB staining for the proteins expressed in *E. coli*. Lower panel is a Western blotting against the Flag-tag of the target proteins. Lanes without labeling are irrelevant to the text.

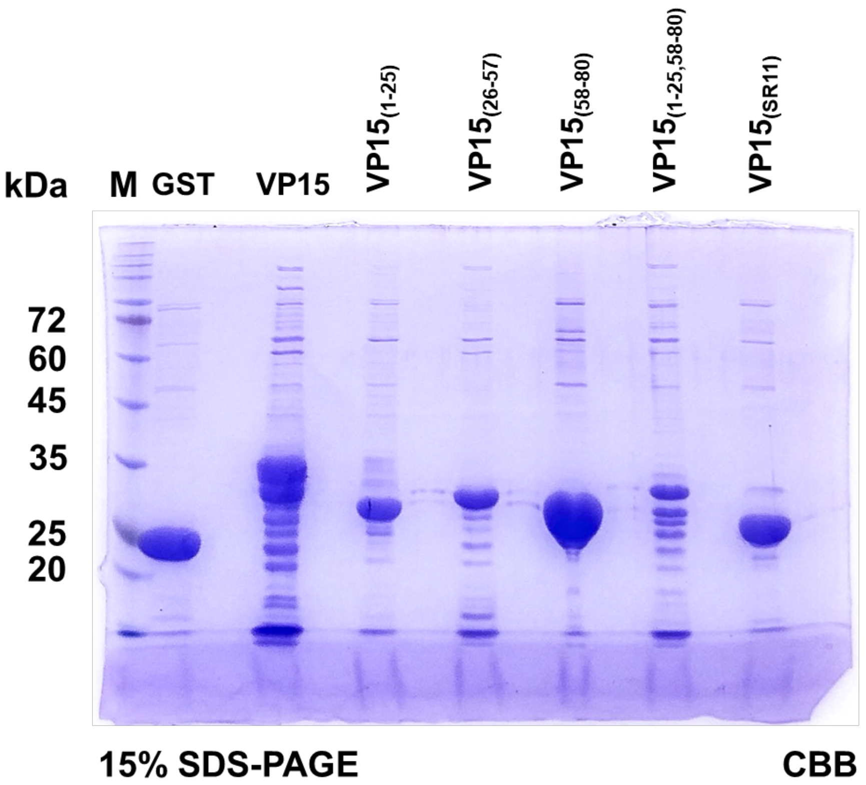

Figure S3. SDS-PAGE analysis of the purified proteins. This is the original image for Figure 1E.

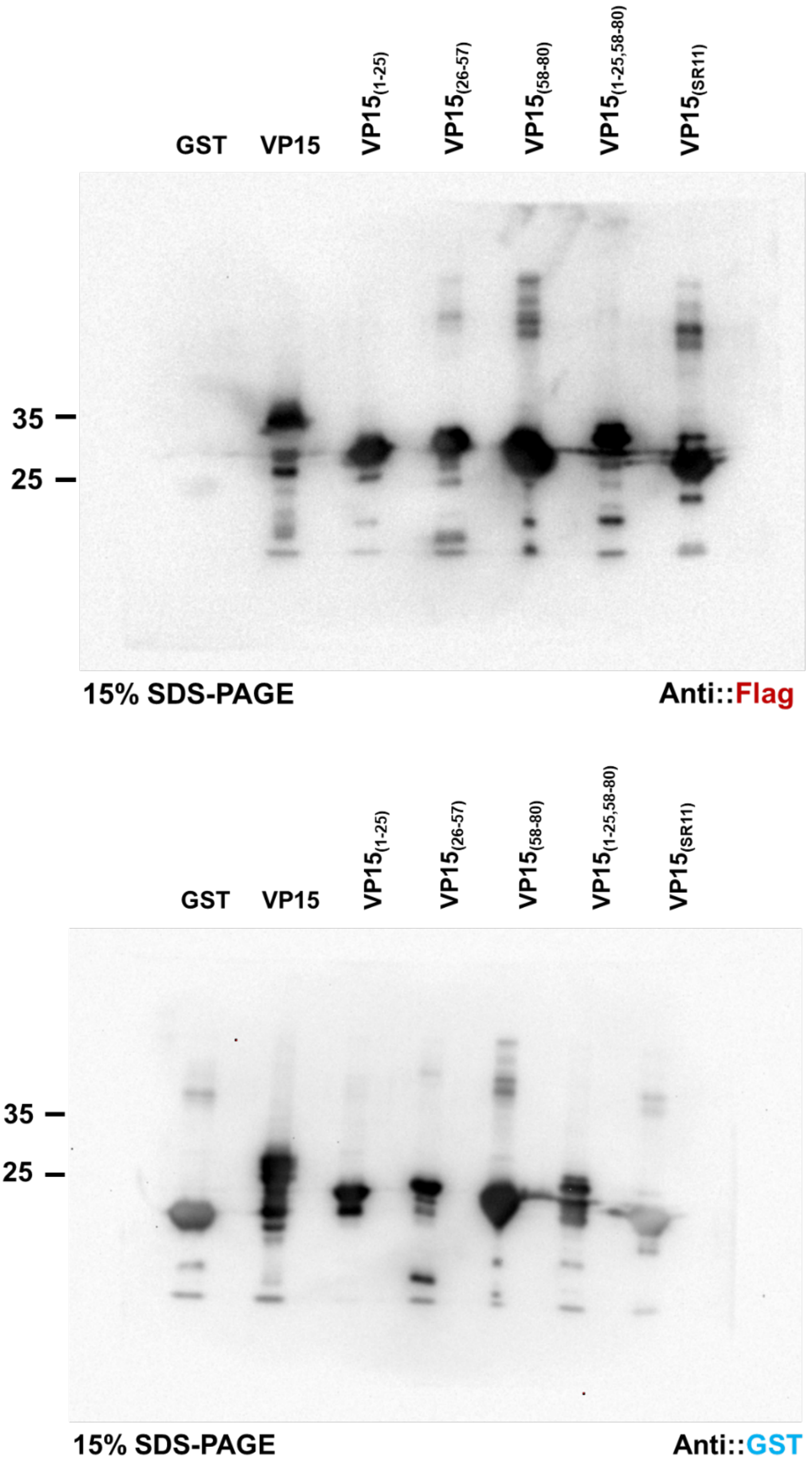

Figure S4. Western blot analysis of the purified proteins using anti-Flag and anti-GST antibodies. The proteins were purified from *E. coli* using GST affinity chromatographic column and the purified recombinant products were confirmed using Western blot analysis. These are the supplements for Figure 1E.

1 cgtctgcagttcacgtgtacgagaggcgaaggagacactcagctgcctcgctttcccccc

61 cgattcatacagtttccagagccgactcactaaagaccATGAGTGCCATCAGTCGTGCTA

1 M S A I S R A

121 TGTACCGCCTGCAAGGCTCCCTGCTGCCAGGAATAATGGGCGTTCGGGGGATCGTGCCAC

8 M Y R L Q G S L L P G I M G V R G I V P

181 CGTCGCGGAACCTGACGAGGAACTTGGTGGTGATGTGTTCAAACGCCGGTCATGGCCGCC

28 P S R N L T R N L V V M C S N A G H G R

241 GCTCGCAGCTCCAGTCCTCCACGCTGTGTTCCTGCGGCTGCGGCATCCATGGCATGCACA

48 R S Q L Q S S T L C S C G C G I H G M H

301 CGAGAGGAGATAGAGAGCTGGTAGAATTCCTACAGGAGGAAATAGTAGCTGAGAAGAAGA

68 T R G D R E L V E F L Q E E I V A E K K

361 CTATGGCCAGTGGCGTTCCTTCACATTTAGATGATTTCAAAATTAGTGTTAATGATGCAG

88 T M A S G V P S H L D D F K I S V N D A

421 AAGTCACTCTCACAAAGAATTTCCATGATGAAGTCATCACCATCAGCCTCAACGTGAACC

108 E V T L T K N F H D E V I T I S L N V N

481 ACACTGTAGACACCGACGCCCCAGCGGAGATGAGTCAAGACCAATCTGAGGGAGAGCTCA

128 H T V D T D A P A E M S Q D Q S E G E L

541 AGAGTCGTCCCAGCTTTGAAGTTGACATCAAGGTTGGCTCAAAGACCCTGTCCTTCACAT

148 K S R P S F E V D I K V G S K T L S F T

601 GCTCATACACAGGGCCAAGTGATATTACAGAAGGTCAAGACCAGGTGGACGATGTTTTTG

168 C S Y T G P S D I T E G Q D Q V D D V F

661 GTATCAATGAGCTTACCATGTACGAGGGAGAATGGAATGAAGAAGTCTACTGTGTTTCAG

188 G I N E L T M Y E G E W N E E V Y C V S

721 GAGATATTTTGGATGGTATGATGTATGACCTTCTGATGAATATGTTGGAGGAGAGAGGAG

208 G D I L D G M M Y D L L M N M L E E R G

781 TCACAAATGAATTCGCAGACAAGCTGAGCAATGTCTGCTCGGACTATGAGCATTATATGT

228 V T N E F A D K L S N V C S D Y E H Y M

841 ACGTTAACTTGCTTCAAAAAGTACAGGACTTTGTCAAGAGGAAATAAatgggaaacaaga

248 Y V N L L Q K V Q D F V K R K *

901 caatcacaatttcatcaaaccccaagtttcaagttagagggatggtctgccttgaaaact

961 taaccattcatgttcattctaaatatgtatgtattggatatggttcctgt***attta***gtttg

1021 acaattgaagcttcttttttttgtgatataaagtcaaattttgatcattcataccacaag

1081 aacattacaaactttatatgttcatagtttgttttatgttatacaagttatgtttatatt

1141 gactgaggaatggtattgtctttgcagaaggcaagacagtgtaattaatatgtacagaag

1201 aaacctagtttatatgttca

Figure S5. The nucleotide and amino acid sequence of MjgC1qR. The start codon (ATG) is underlined. The stop codon is marked by a black asterisk. The mitochondrial cleavage site is indicated by red characters, and the mitochondrial acidic matrix protein domain (MAM33) is underlined in blue color. The RGD domain is labeled in green characters. mRNA instability element (ATTTA) is indicated by bold italic.

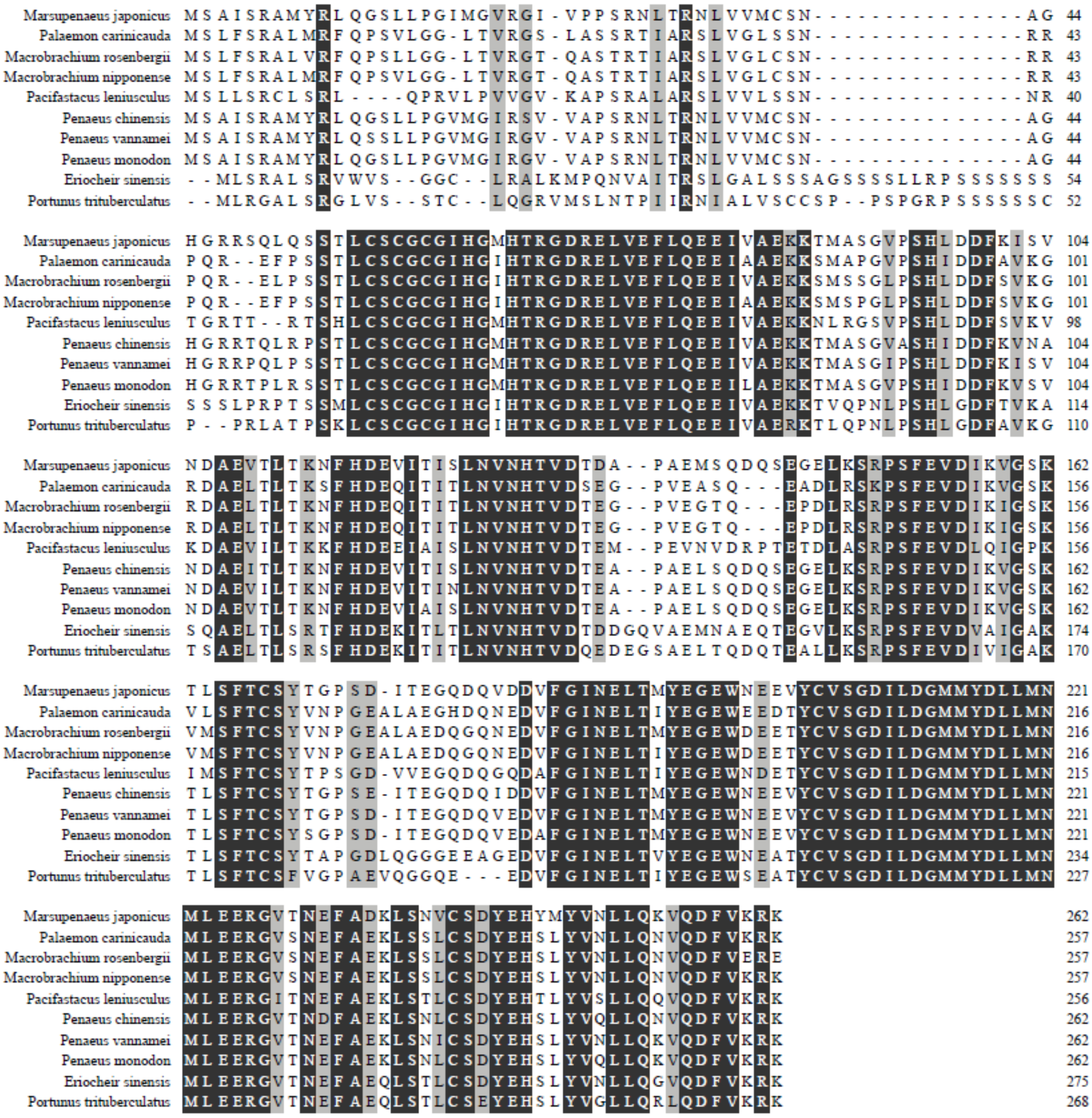

Figure S6. Multiple alignments of the predicted amino acid sequence of MjgC1qR with other gC1qR(s) using Clustal omega software. Consensus amino acids are shaded using Multiple Align Show (Black: identical residues, Grey: similar residues). The accession numbers of the related sequences are as follow: *P. carinicauda* (AFY05651.1), *M. rosenbergii* (AJE28353.1), *M. nipponense* (QEU52623.1), *P. leniusculus* (AEC50078.1), *P. chinensis* (AFJ59951.1), *P. vannamei* (AGO21477.1), *P. monodon* (ADV18978.1), *E. sinensis* (ANN46490.1) and *P. trituberculatus* (AYV97197.1).

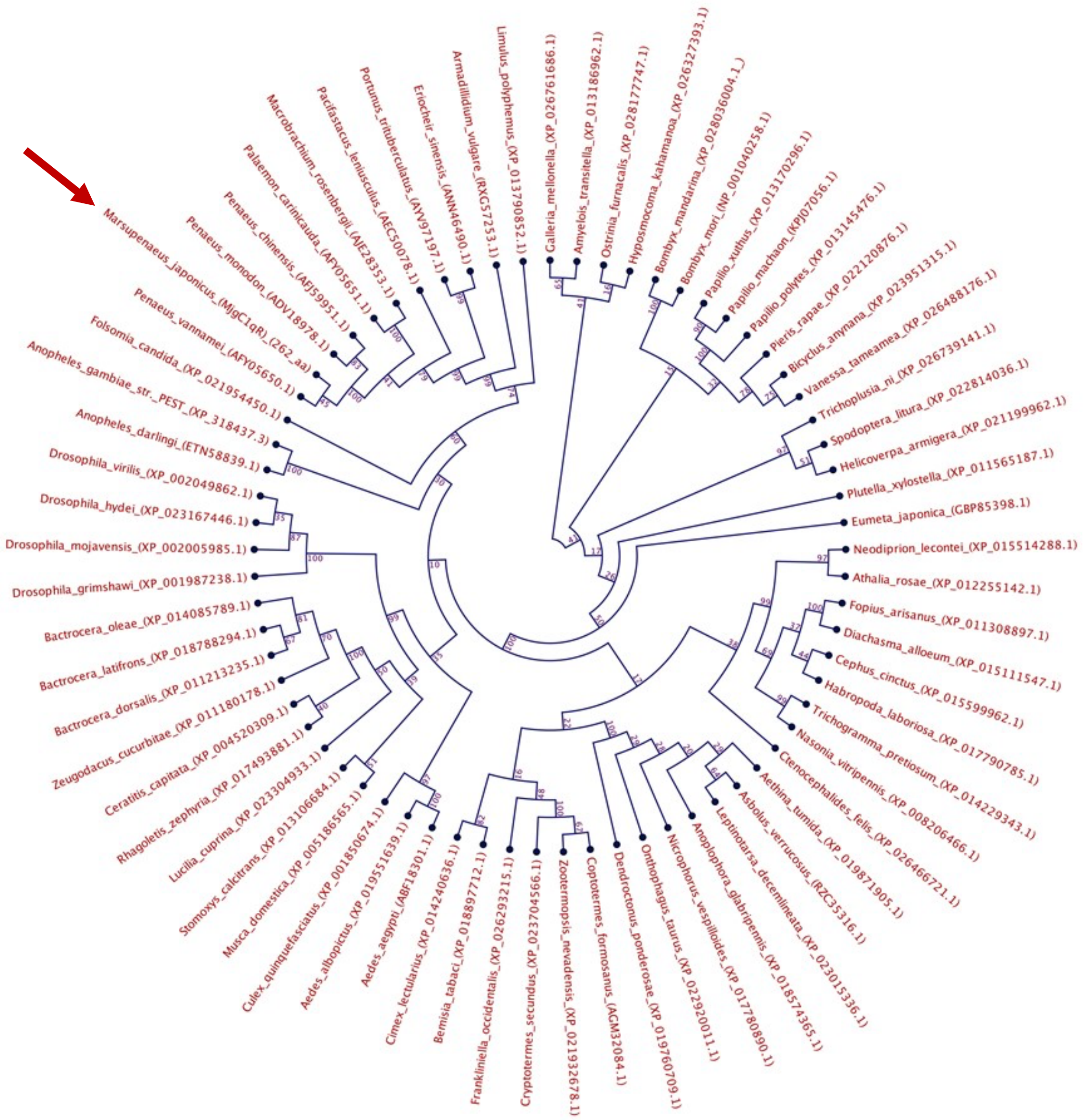

Figure S7. Phylogenetic tree generated based on the amino acid of gC1qR from different species. Accession numbers are shown in the figure.

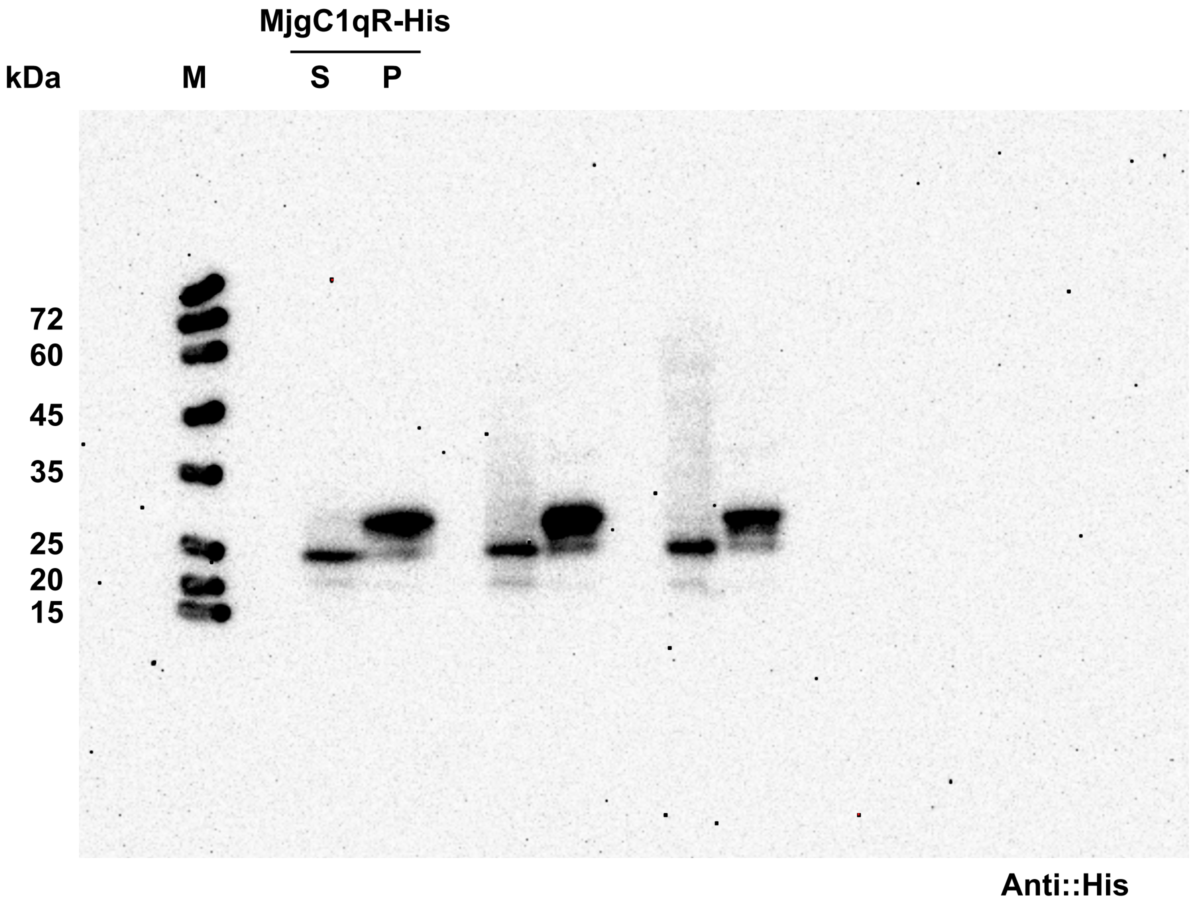

Figure S8. The original full-length blot for Figure 4.

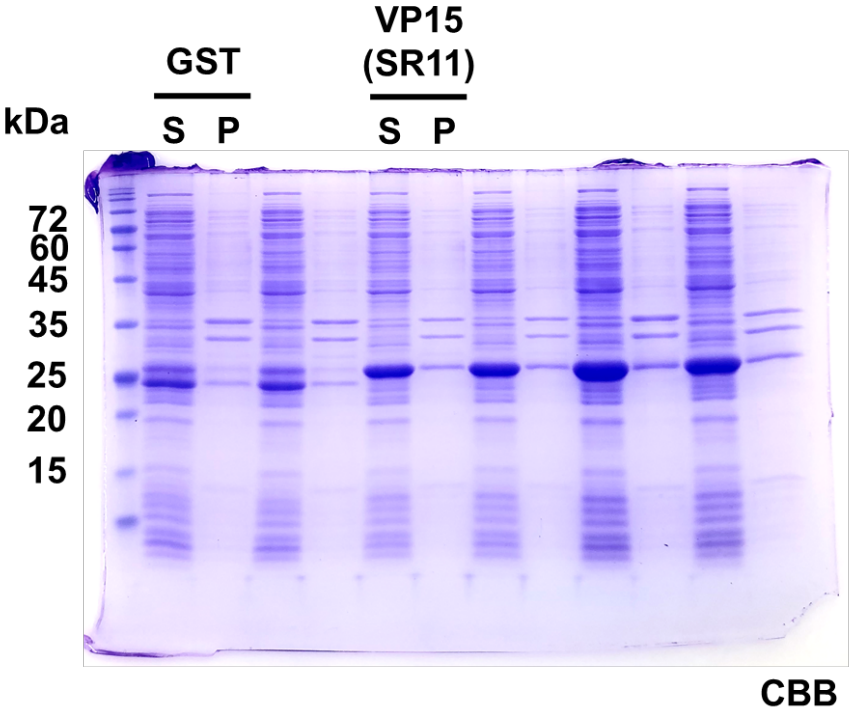

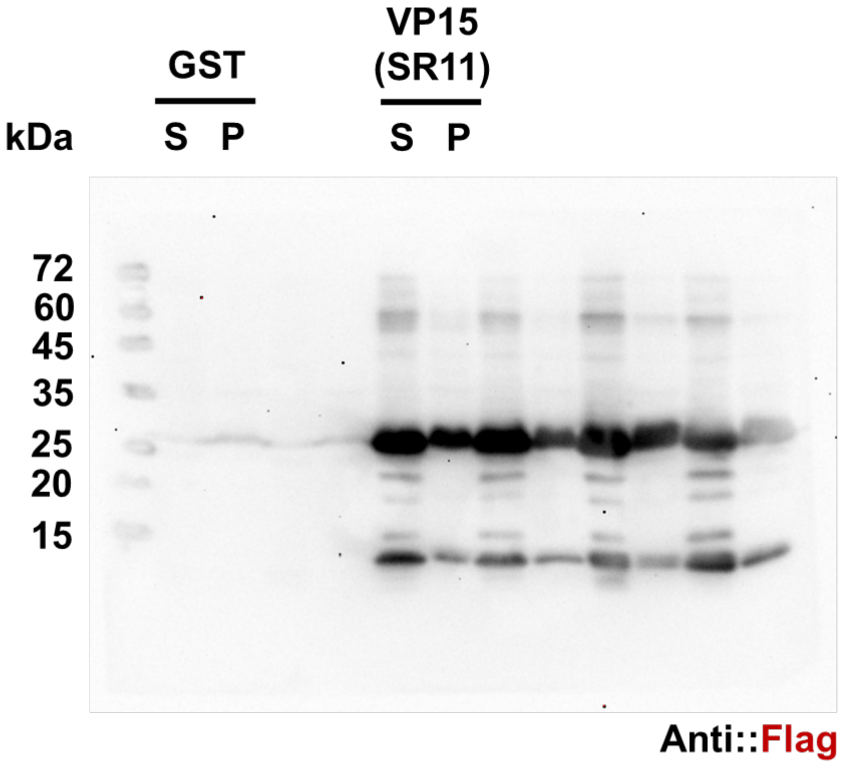

Figure S9. Expression of the GST-SR11 using the *E. coli* expression system. (Up) Coomassie brilliant blue-stained SDS-PAGE gel of *E. coli* expressing GST-SR11 and (Down) Western blot analysis of the expressed recombinant protein using anti-Flag antibody.

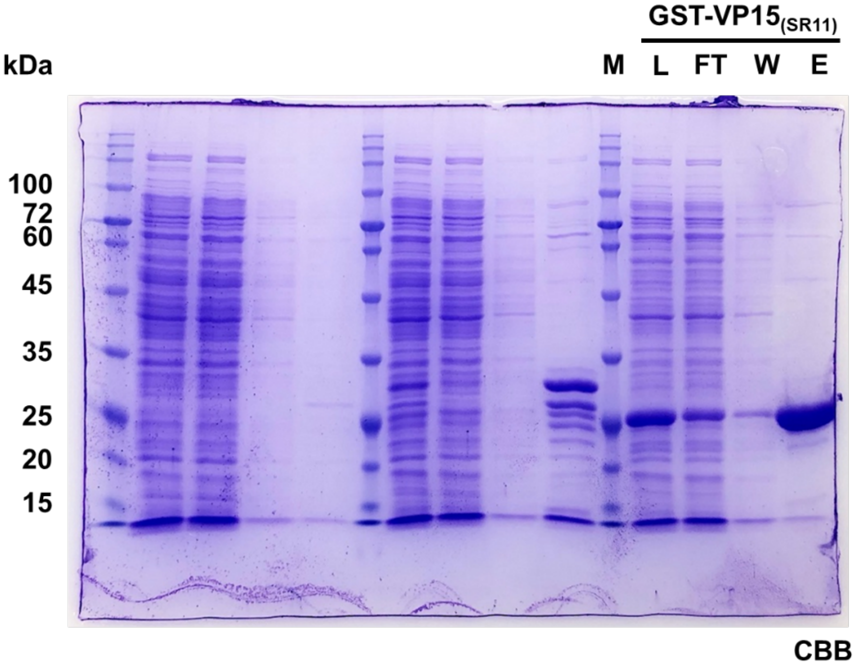

Figure S10. Purification of GST-SR11 using GST affinity column. M: marker, L: loading, FT: flow through, and E: elution.

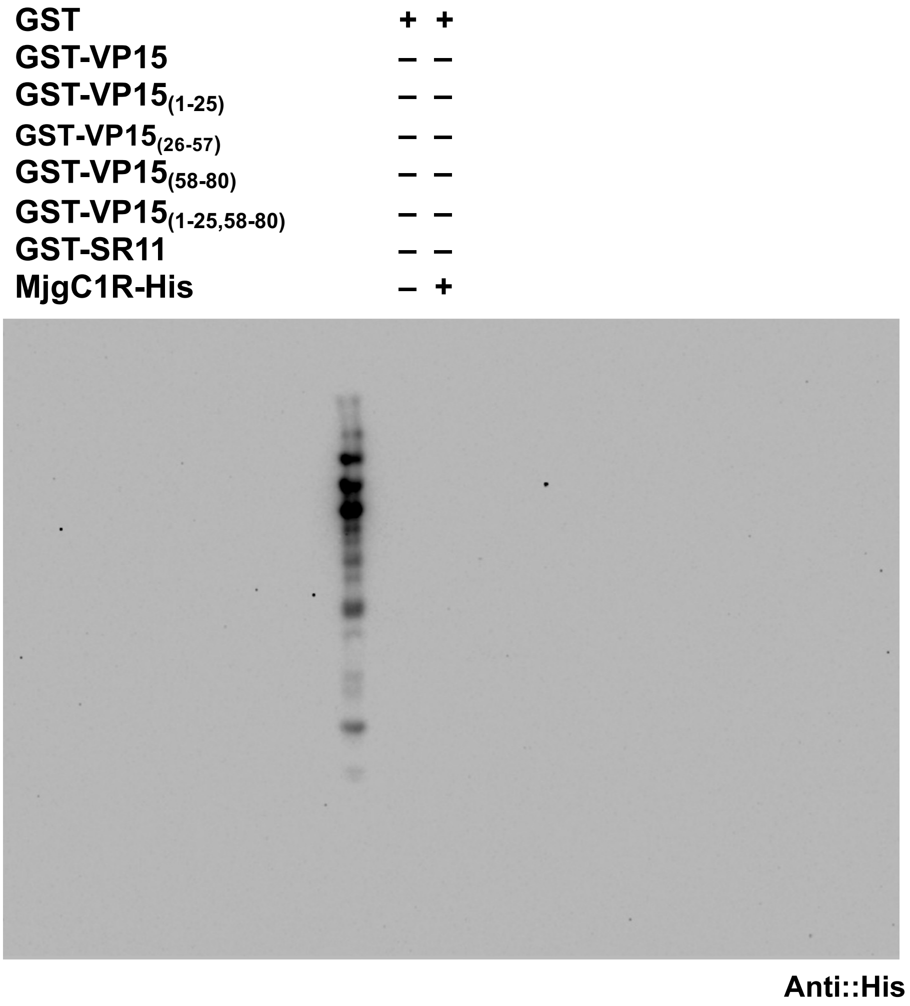

Figure S11. The original full-length blots for Figure 5A-1 of GST pull-down assay. Western blot analysis against the His-tag of recombinant MjgC1qR.

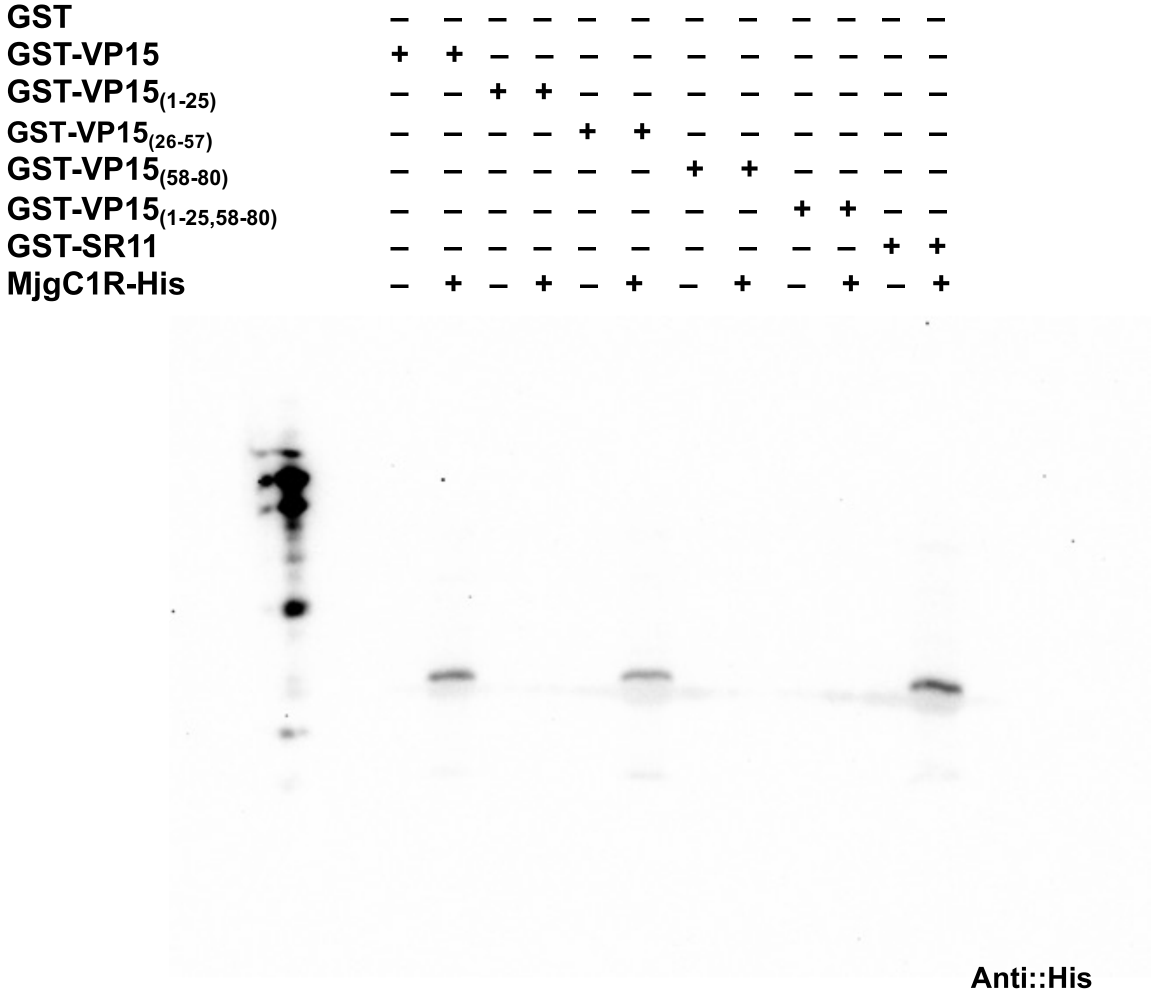

Figure S12. The original full-length blots for Figure 5A-2 of GST pull-down assay. Western blot analysis against GST-fused VP15, VP15$_{(1-25)}$, VP15$_{(26-57)}$, VP15$_{(58-80)}$, and VP15$_{(1-25,58-80)}$ and SR11 using anti-His antibody.

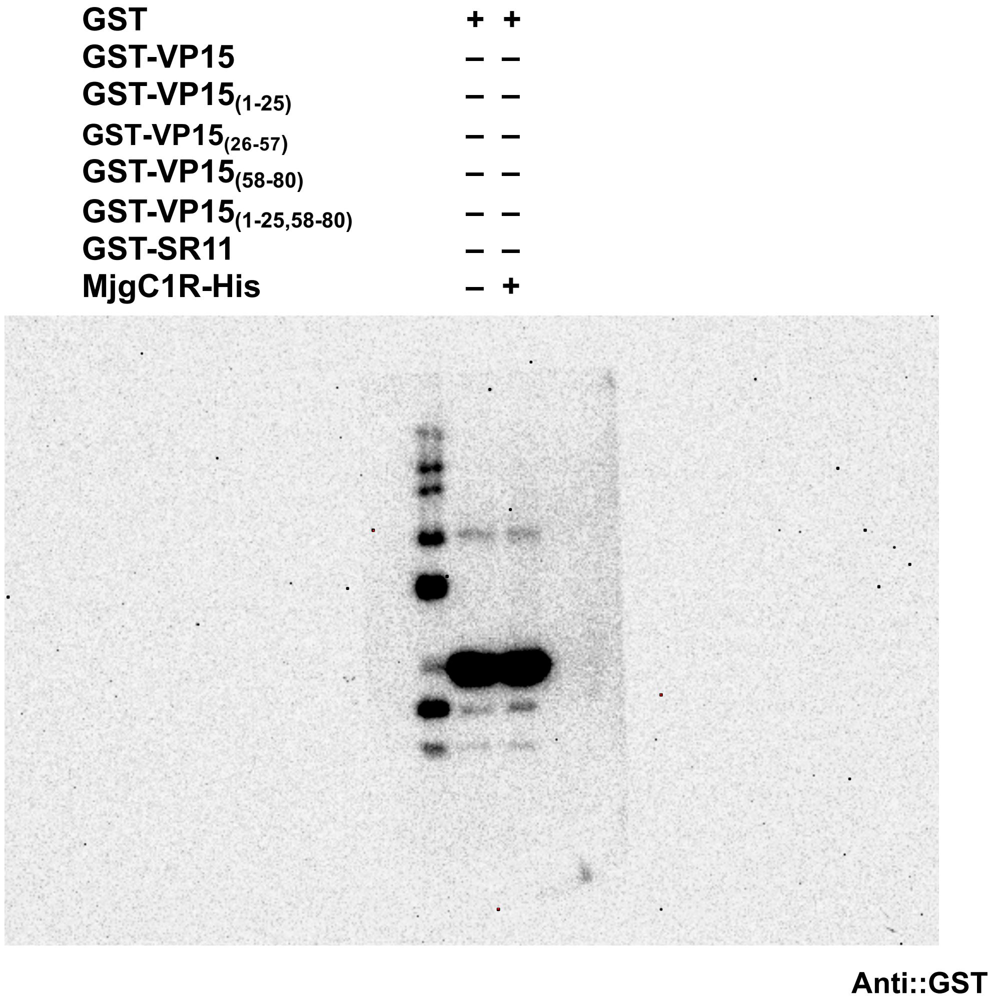

Figure S13. The original full-length Western blots of GST pull-down assay with anti-GST antibody of Figure 5A-3.

**GST**	–	–	–	–	–	–	–	–	–	–	–	–
**GST-VP15**	+	+	–	–	–	–	–	–	–	–	–	–
**GST-VP15$_{(1\text{-}25)}$**	–	–	+	+	–	–	–	–	–	–	–	–
**GST-VP15$_{(26\text{-}57)}$**	–	–	–	–	+	+	–	–	–	–	–	–
**GST-VP15$_{(58\text{-}80)}$**	–	–	–	–	–	–	+	+	–	–	–	–
**GST-VP15$_{(1\text{-}25,58\text{-}80)}$**	–	–	–	–	–	–	–	–	+	+	–	–
**GST-SR11**	–	–	–	–	–	–	–	–	–	–	+	+
**MjgC1R-His**	–	+	–	+	–	+	–	+	–	+	–	+

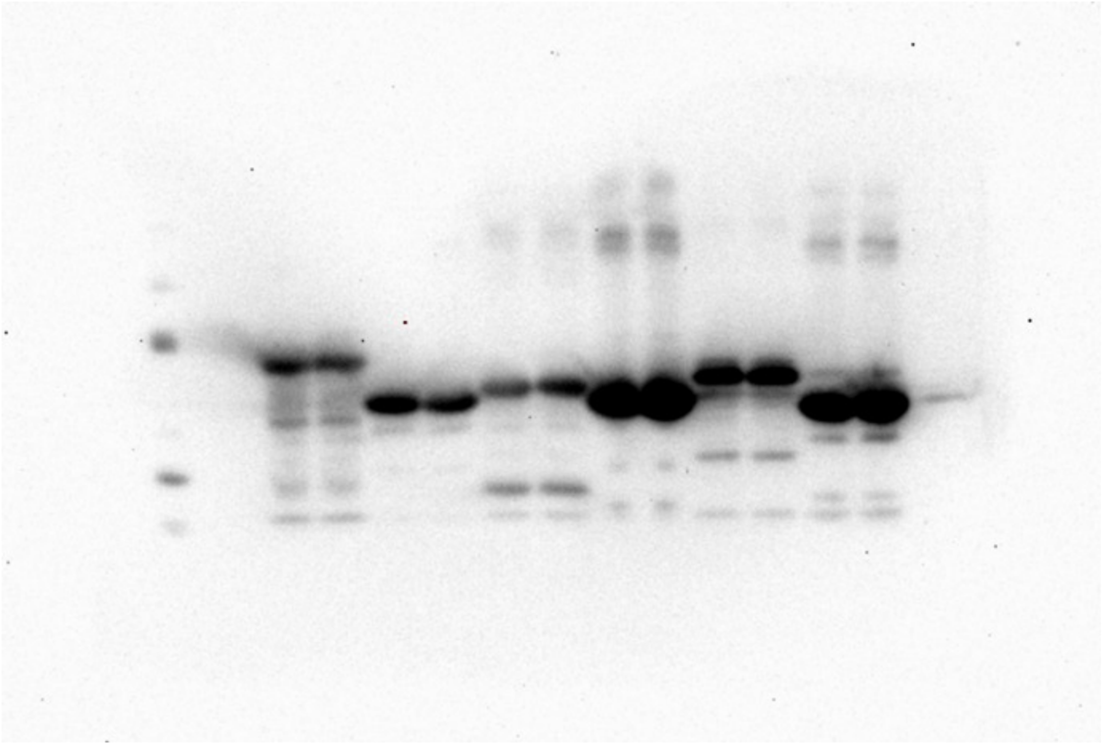

Figure S14. The original full-length blots for Figure 5A-4 of GST pull-down assay. Western blot analysis against GST-fused VP15, VP15$_{(1\text{-}25)}$, VP15$_{(26\text{-}57)}$, VP15$_{(58\text{-}80)}$, and VP15$_{(1\text{-}25,58\text{-}80)}$ and SR11 using anti-Flag antibody.
